# Circulating fatty acids and osteoarthritis: evidence from observational and genetic analyses

**DOI:** 10.1017/S0007114526106291

**Published:** 2026-06-14

**Authors:** Jinyu Zhou, Xunying Zhao, Tao Han, Linna Sha, Rong Xiang, Bowen Lei, Jiangbo Zhu, Yanqiu Zou, Zhixin Tan, Yang Qu, Jiaojiao Hou, Qin Deng, Sirui Zheng, Ting Yu, Xiaofeng Ma, Xin Song, Bin Yang, Di Zhang, Mengyu Fan, Xia Jiang

**Affiliations:** 1 Department of Nutrition and Food Hygiene, West China School of Public Health and West China Fourth Hospital, Sichuan University, Chengdu, People’s Republic of China; 2 Department of Epidemiology and Biostatistics and West China-PUMC C. C. Chen Institute of Health, West China School of Public Health and West China Fourth Hospital, Sichuan University, Chengdu, Sichuan, People’s Republic of China; 3 Department of Clinical Neuroscience, Center for Molecular Medicine, Karolinska Institutet, Solna, Stockholm, Sweden

**Keywords:** Fatty acids, Osteoarthritis, Causal inference, Genetic correlation, Pleiotropic loci

## Abstract

Dysregulation of fatty acids metabolism has been associated with the risk of osteoarthritis (OA), yet current evidence from epidemiological or genetic studies remains inconclusive. We aimed to investigate the phenotypic association and genetic architecture between total fatty acids, saturated fatty acids (SFA), MUFA, PUFA and OA. Leveraging individual-level data from the UK Biobank, combined with the hitherto largest genome-wide association studies of fatty acids (*n* 136 016) and OA (*n* 826 690) in European individuals, we implemented a comprehensive analytical framework. This included observational and genetic analyses, incorporating phenotypic associations, genetic correlations, cross-trait meta-analysis, enrichment analysis and Mendelian randomisation (MR). Observational analysis identified SFA as a risk factor, while MUFA and PUFA as protective factors for OA. Despite a lack of genome-wide genetic correlation, statistically significant local signals were detected within three specific genomic regions. Cross-trait meta-analysis identified sixty-eight pleiotropic loci shared between fatty acids and OA, of which nine were novel. Enrichment analysis revealed the shared genes were enriched in lipoprotein metabolism, immune response and inflammation regulation pathways. Two-sample MR provided evidence for a causal relationship of MUFA and PUFA on OA that survived false discovery rate correction. This study supports associations between circulating fatty acids and OA, with MUFA and PUFA exerting a protective role. Our findings provide new perspectives into OA prevention especially regarding the potential dietary interventions.

Osteoarthritis (OA) is a chronic degenerative joint disorder characterised by progressive cartilage degradation, subchondral bone remodelling and synovial inflammation, affecting over 500 million individuals worldwide with substantial socio-economic burden^(1–3)^. Growing evidence suggests that metabolic dysregulation, particularly in fatty acids, plays a crucial role in OA pathogenesis^(4,5)^. Mechanistically, certain fatty acids, such as long-chain *n*-3 PUFA, can influence OA progression by modulating inflammatory mediators, oxidative stress and cartilage matrix metabolism in joint tissues^(4,6)^.

Nevertheless, epidemiological research has predominantly centred on self-reported dietary intake^(7,8)^, leaving scarce and inconsistent evidence on circulating fatty acids, and largely overlooking the potential non-linear relationships. While one cross-sectional study in knee OA patients reported a link between elevated *n*-3 PUFA levels and reduced patellofemoral damage severity^(9)^, another study found no significant association of PUFA with either radiographic OA or symptomatic OA^(10)^. These discrepancies may stem from study design and methodological limitations such as follow-up durations, confounders and reverse causation.

Leveraging genetic insights offers a promising strategy to unravel these complexities. Twin studies have demonstrated substantial heritability for both fatty acids (20–60 %)^(11)^ and OA (39–65 %)^(12,13)^, providing a foundation for genetic epidemiological investigations. In fact, recent research has attempted to explore causal relationships between fatty acids and OA using a Mendelian randomisation (MR) design. Two studies examining MUFA and OA in European populations of less than 10 000 individuals found no association^(14,15)^, while a larger study of 114 999 Europeans suggested that PUFA may reduce the risk of knee and hip OA^(16)^. These MR analyses were limited by small sample sizes or reliance on early genome-wide association studies (GWAS) datasets, imbalanced case–control ratios, lack of individual-level data and ignorance of non-linear effects. Furthermore, they focused solely on vertical pleiotropy, neglecting potential horizontal pleiotropy across complex traits.

Considering these gaps, we conducted a comprehensive investigation to examine the phenotypic association and genetic architecture between circulating fatty acids and OA. Our study employed observational epidemiological methods to evaluate phenotypic associations and potential dose–response relationships, as well as genetic analyses to quantify genetic overlap and identify pleiotropic loci. To further elucidate the relationship, we performed enrichment analysis for biological functions and applied MR, including both one-sample and two-sample designs, to assess causal effects. An overview of our study design is presented in Figure 1.

**Figure 1.**
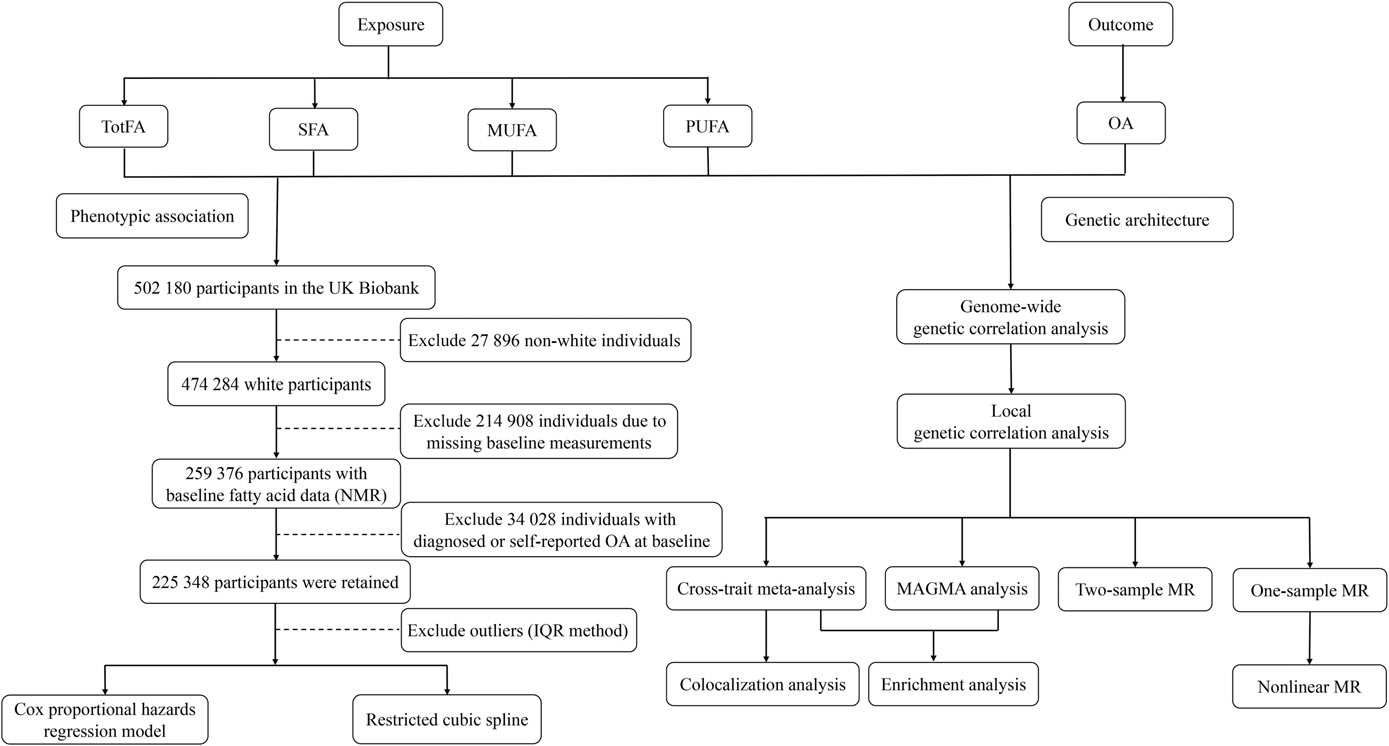
Figure 1 long description. Flowchart of overall study design in European ancestry individuals.

## Materials and methods

### UK Biobank data

The UK Biobank (UKB) is a large-scale prospective cohort study comprising over 500 000 participants aged 40–69 years^(17)^. It has collected extensive phenotypic and genotypic data, including questionnaire responses, physical measurements, sample assays, genome-wide genotyping and longitudinal follow-up for health-related outcomes. Fatty acid levels (measured in mmol/L) were quantified using nuclear magnetic resonance spectroscopy. OA diagnoses were defined based on first occurrences codes M15-M19, with data sourced from primary care records, hospital inpatient data, death registries and self-reported information. We excluded participants with diagnosed or self-reported OA at baseline, as well as those identified as outliers according to the interquartile range method. The study included only participants of White European ancestry who had complete data on fatty acids and relevant covariates.

### Genome-wide association studies summary data

#### Fatty acids genome-wide association studies

We utilised the hitherto largest GWAS of fatty acids, conducted by Karjalainen *et al.* in 2024, which meta-analysed data from thirty-three studies comprising 136 016 participants (88·4 % of European ancestry)^(18)^. Fatty acids were quantified using nuclear magnetic resonance spectroscopy by evaluating specific peaks corresponding to their characteristic chemical shifts. We focused on four main categories of circulating fatty acids: total fatty acids (TotFA), SFA, MUFA and PUFA.

#### Osteoarthritis genome-wide association studies

We utilised the GWAS of OA from a meta-analysis of twenty-onw cohorts conducted by the Genetics of Osteoarthritis consortium, comprising 826 690 individuals (93·4 % of European ancestry)^(19)^. OA cases were defined based on self-reported data, clinical diagnoses, ICD-10 codes (M15-M19, M47·2, M47·8 and M47·9) and radiographic data. We used OA at any site as the outcome, consisting of 177 517 cases and 649 173 controls.

The details of each included GWAS are summarised in online Supplementary Table 1.

### Statistical analysis

#### Observational analysis

Descriptive statistics were conducted to characterise the baseline UKB participants. Continuous variables were summarised as means and standard deviations (Mean (sd)), while categorical variables were presented as frequencies and proportions (*n*, %). We constructed a Cox proportional hazards regression model with baseline measured fatty acids as the exposure. We implemented two models: a basic model adjusting for age, sex, assessment centre, BMI, genotype batch and ten principal components and a fully adjusted model that additionally controlled for education, smoking status, alcohol drinker status, Townsend deprivation index and and mutual adjustment among fatty acids (excluding TotFA from mutual adjustment to avoid collinearity). Restricted cubic spline analysis was applied to evaluate the dose–response relationship between fatty acids and the risk of OA. A two-sided *P* value of less than 0·05 was considered statistically significant.

#### Overall and local genetic correlation analyses

To estimate genome-wide genetic correlation between pairs of traits, we performed a high-definition likelihood method by fully accounting for linkage disequilibrium across the genome^(20)^. Genetic correlation ( $r_{g}$ ) ranges from −1 to 1, with −1 indicating a perfect negative correlation and 1 indicating a perfect positive correlation. A *P* value of less than 0·05 was considered statistically significant.

We also conducted local genetic correlation analysis using SUPER GeNetic cOVariance Analyzer^(21)^. Instead of estimating the average correlation of genetic effects across the genome, SUPER GeNetic cOVariance Analyzer quantifies the genetic similarity of two traits in specific genomic regions. In this analysis, the genome was partitioned into 2353 LD-independent regions with an average size of 1·6 centimorgans. Statistical significance was determined using a Bonferroni correction, with the threshold set at *P* < 0·05/2353.

#### Cross-trait meta-analysis

We conducted a cross-trait meta-analysis using Pleiotropic Locus Exploration and Interpretation using Optimal test (PLEIO) to identify pleiotropic loci^(22)^. PLEIO is an approach that models genetic correlations, heritability and environmental correlations across traits to map pleiotropic loci with GWAS summary statistics.

We identified independent SNP using PLINK’s clumping function (parameters: --clump-p1 5e-8 --clump-p2 1e-5 --clump-r2 0·01 --clump-kb 500)^(23)^. Significant pleiotropic SNP were defined as those satisfying *P*_PLEIO_ < 5 × 10^–8^ and *P*_single-trait_ < 1 × 10^–3^ (for both traits). Novel pleiotropic SNP, which we were particularly interested in, were defined as significant pleiotropic SNP that did not reach genome-wide significance in any single trait and were not in linkage disequilibrium with previously identified SNP related to fatty acids or OA.

The Ensembl Variant Effect Predictor^(24)^ and 3DSNP^(25)^ were used to map these SNP to genes. Variant Effect Predictor identifies candidate genes based on simple physical proximity, while 3DSNP links SNP to their three-dimensional interacting genes.

#### Colocalisation analysis

We performed colocalisation analysis using Coloc to evaluate whether the pleiotropic SNP identified by PLEIO are shared causal variants^(26)^. Coloc applies a Bayesian framework to estimate the posterior probabilities for five hypotheses: H0 (no association with either trait), H1 (association with trait 1, not with trait 2), H2 (association with trait 2, not with trait 1), H3 (association with both traits, distinct casual variants) and H4 (association with both traits, shared casual variant). We focused primarily on the posterior probability of H4 (PPH4), where a PPH4 greater than 0·5 suggests that the SNP is the shared causal variant regulating both traits. The analysis was based on the European-ancestry subset of the 1000 Genomes Project Phase 3 as the linkage disequilibrium reference panel, within a ± 500 kb window.

#### Gene-based analysis

We conducted gene-based analysis using MAGMA to identify candidate genes associated with both traits^(27)^. First, we annotated the SNP included in GWAS, mapping to their corresponding genes based on chromosomal positions. Subsequently, a gene-based analysis was conducted using the European population data from the 1000 Genomes Project, provided by MAGMA as the linkage disequilibrium reference. Finally, we identified a set of significantly associated genes (*P* < 1 × 10^–3^) for each trait, along with the genes shared trait pairs. These shared genes can be classified into three groups based on whether they have been previously reported: known genes in both traits, known genes in a single trait and novel shared genes.

#### Enrichment analysis

To elucidate the biological implications of pleiotropic genes identified through PLEIO and MAGMA, we performed enrichment analyses using Gene Ontology (GO) and Kyoto Encyclopedia of Genes and Genomes (KEGG) by WebGestalt^(28)^. We investigated the shared molecular mechanisms potentially linking fatty acid metabolism to OA development. The enriched GO or KEGG pathways were considered statistically significant when their false discovery rate (FDR)-adjusted *P* values were below 0·05.

#### Mendelian randomisation analysis

We conducted both one-sample and two-sample MR to assess causal relationships between fatty acids and OA. As instrumental variables (IV), we utilised independent genome-wide significant SNP (*P* < 5 × 10^–8^) identified in the original GWAS: forty-five SNP for TotFA, thirty-eight for SFA, forty-five for MUFA and fifty-five for PUFA (online Supplementary Tables 14–17)^(18)^. For all IV, both per-SNP and Sanderson–Windmeijer conditional F-statistics substantially exceeded 10.

For two-sample MR, we employed the random-effect inverse-variance weighted method as the primary approach. Sensitivity analyses were performed using weighted median approach, MR-Egger regression, MR-Pleiotropy Residual Sum and Outlier, as well as a repeat inverse-variance weighted analysis after removing pleiotropic SNP^(29–31)^. A causal effect was considered significant if the IVW *P* value was < 0·05 after FDR correction, and estimates from the other methods were directionally consistent.

For one-sample MR, we constructed weighted polygenic risk scores using the GWAS of fatty acids. Two linear MR models (basic and fully adjusted) were applied, followed by non-linear MR stratifying participants into quartiles based on residual fatty acid levels, with separate analyses conducted within each quartile^(32)^. We assessed heterogeneity using Cochran’s *Q* statistic and examined non-linearity through meta-regression of MR estimates against the mean fatty acid levels in each quartile.

## Results

### Observational analysis

**Figure 2.**
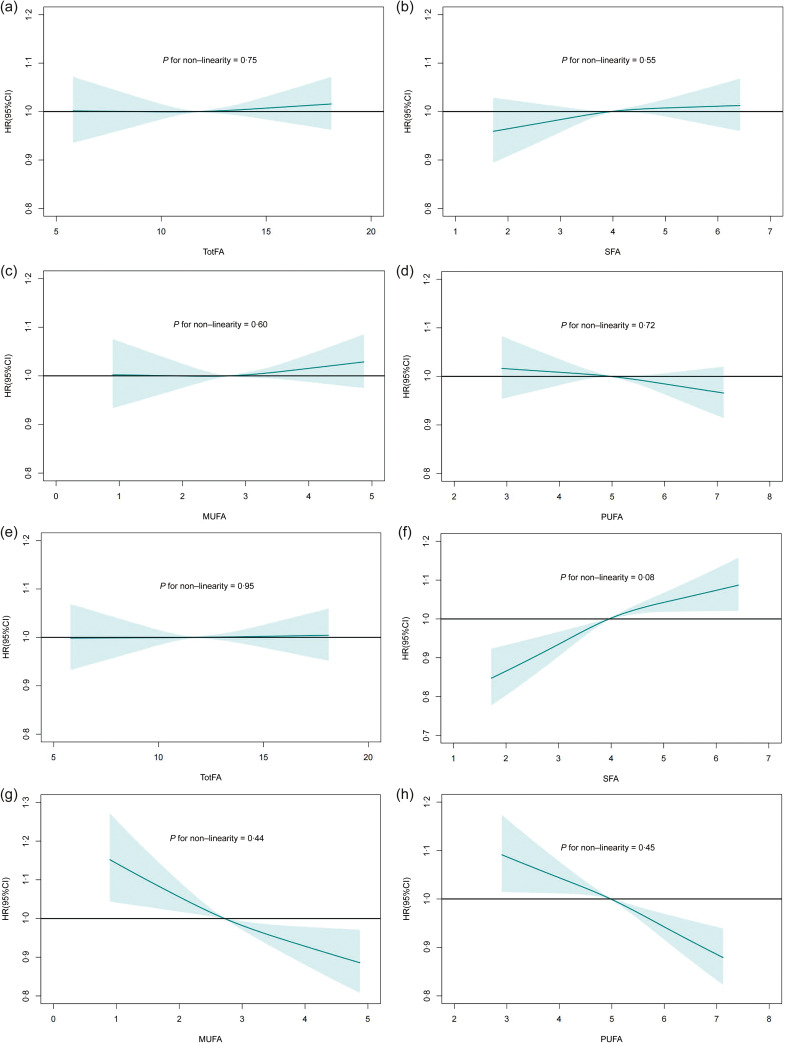
Analysis of restricted cubic spline regression. (a) Relationship between TotFA and OA in Model 1. (b) Relationship between SFA and OA in Model 1. (c) Relationship between MUFA and OA in Model 1. (d) Relationship between PUFA and OA in Model 1. (e) Relationship between TotFA and OA in Model 2. (f) Relationship between SFA and OA in Model 2. (g) Relationship between MUFA and OA in Model 2. (h) Relationship between PUFA and OA in Model 2. Solid lines represent the estimated regression coefficients, while the shaded green areas indicate the 95 % confidence intervals. OA, osteoarthritis; TotFA.

The baseline characteristics of UKB participants included in the observational analysis are presented in online Supplementary Tables 2–5. In the basic model, no significant association was found between any fatty acid and OA (TotFA: *P* = 0·59, SFA: *P* = 0·13, MUFA: *P* = 0·30 and PUFA: *P* = 0·09, Table 1). However, after further controlling for common confounders, a risk effect of circulating SFA levels (hazard ratio 1·05; 95 % confidence interval 1·03, 1·07) as well as protective effects of MUFA (hazard ratio 0·94; 95 % CI 0·90, 0·98) and PUFA (hazard ratio 0·95; 95 % CI 0·93, 0·97) on OA incident were identified. There was no dose–response relationship of fatty acids with OA according to the restricted cubic spline analysis (Figure 2).

**Table 1.** Observational associations between fatty acids and OA Table 1 long description.

	*n*	Cases/Person-years	Model 1		Model 2	
			HR	95 % CI	HR	95 % CI
TotFA	211 161	33 872/2 597 849	1·00	1·00, 1·01	1·00	1·00, 1·01
SFA	209 999	33 606/2 584 079	1·01	1·00, 1·02	1·05	1·03, 1·07
MUFA	209 548	33 553/2 578 721	1·01	1·00, 1·02	0·94	0·90, 0·98
PUFA	212 267	34 142/2 610 743	0·99	0·97, 1·00	0·95	0·93, 0·97

OA, osteoarthritis.

### Overall and local genetic correlations

**Figure 3.**
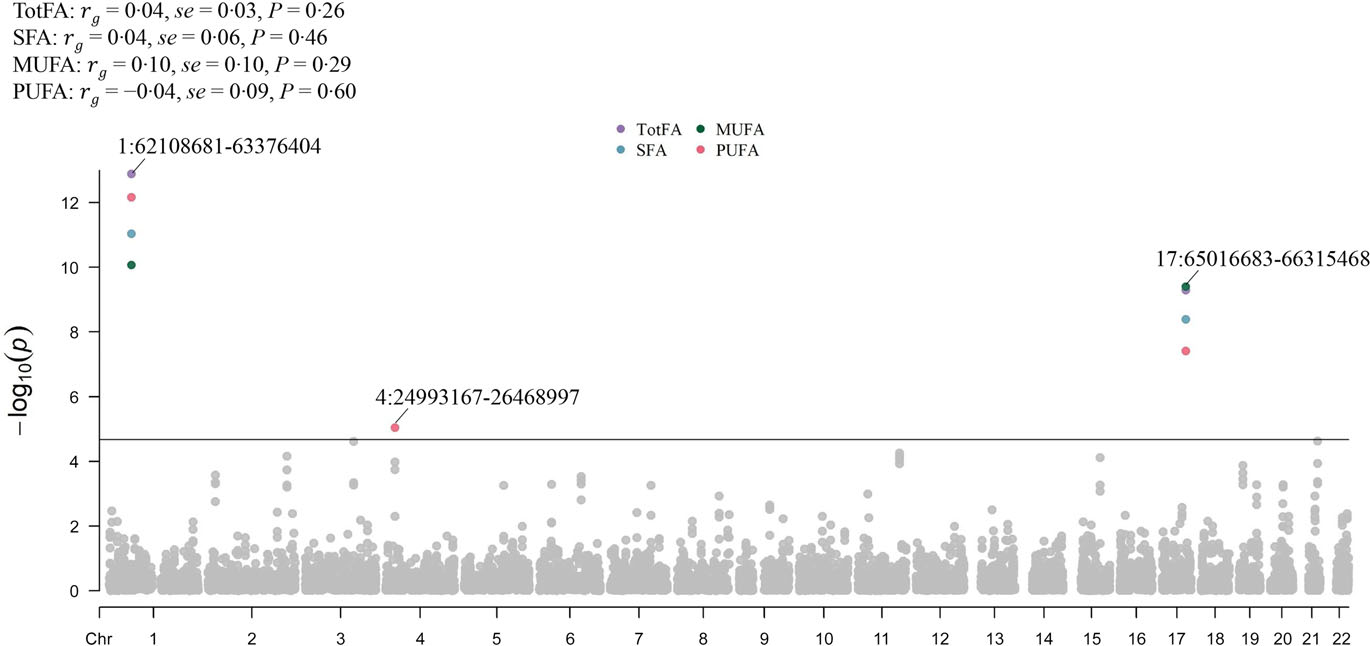
Genome-wide and local genetic correlations between fatty acid and OA. The top-left corner of the figure displays the results from the genome-wide association analysis. In the Manhattan plot, the coloured dots represent loci that are significant for local genetic correlation after multiple testing correction. OA, osteoarthritis.

High-definition likelihood analysis revealed no significant genome-wide genetic correlation between fatty acids and OA (Figure 3). By partitioning the entire genome into specific regions, we identified three non-overlapping local signals, comprising two signals for TotFA, two for SFA, two for MUFA and three for PUFA (Figure 3). Signals at 1p31·3 (chr1:62108681–63376404) and 17q24·2 (chr17:65016683–66315468) were observed across four fatty acids (TotFA, SFA, MUFA and PUFA) and OA, highlighting their importance as shared loci. Notably, the most significant signal for each fatty acid was consistently found in 1p31·3, a region encompassing several key genes, including *USP1* and *DOCK7*, previously implicated in fatty acid metabolism^(33,34)^, as well as *TM2D1*, *PATJ*, *RPS15AP7* and *L1TD1*, linked to BMD according to prior studies^(35–37)^.

### Cross-Trait meta-analysis

**Figure 4.**
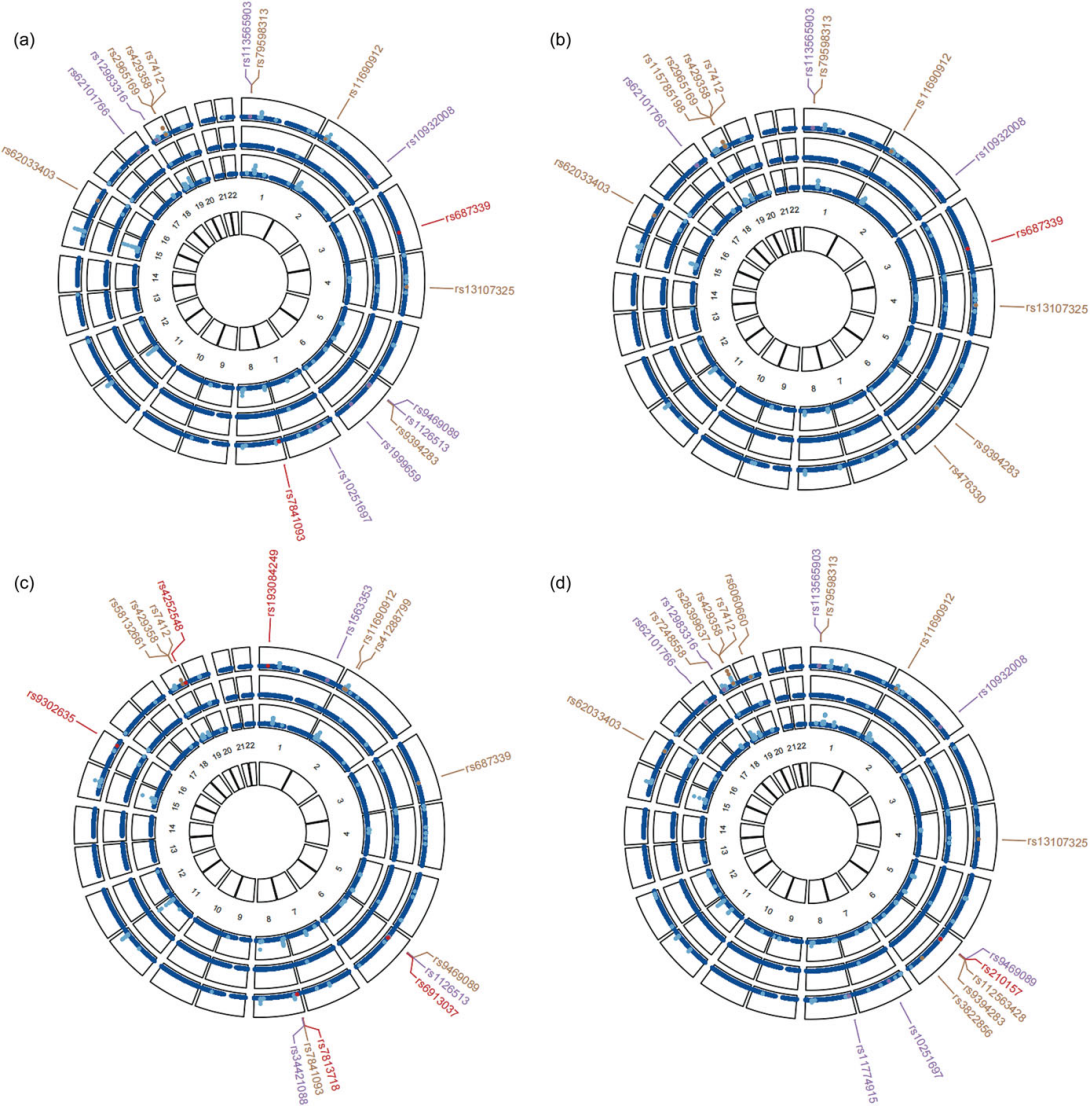
Figure 4 long description. Cross-phenotype association between fatty acid and OA. (a) Circular Manhattan plot between TotFA and OA. The outermost circle shows the cross-trait meta-analysis results; inner circles show GWAS results for TotFA and OA, respectively. Light blue indicates genome-wide significant variants; dark blue indicates non-significant variants. SNP are divided into four different categories according to their single-trait and cross-trait characteristics: single-trait-driven shared SNP (brown), LD-tagged shared SNP (purple) and novel shared SNP (red). Corresponding RS ID are listed. (b) Circular Manhattan plot between SFA and OA. (c) Circular Manhattan plot between MUFA and OA. (d) Circular Manhattan plot between PUFA and OA. OA, osteoarthritis; TotFA, total fatty acids.

Motivated by the observed local genetic correlations, we next conducted cross-trait meta-analysis to detect pleiotropic loci. A total of sixty-eight significant pleiotropic loci were identified for OA with at least one fatty acid, comprising eighteen loci shared with TotFA, fourteen with SFA, sixteen with MUFA and twenty with PUFA (Figure 4, online Supplementary Tables 6–7). Across all pleiotropic loci, the most significant locus was rs429358 at 19q13·32 (*P*_PLEIO_ = 4·45 × 10^–229^ for PUFA-OA), followed by rs429358 at 19q13·32 (*P*_PLEIO_ = 2·12 × 10^–163^) and rs7412 at 19q13·31 (*P*_PLEIO_ = 1·09 × 10^–146^) for TotFA-OA.

Among these sixty-eight pleiotropic loci, we identified nine novel loci, including two shared with TotFA (rs687339 and rs7841093), one with SFA (rs687339), five with MUFA (rs193084249, rs6913037, rs7813718, rs9302635 and rs4252548) and one with PUFA (rs210157) (Figure 4, online Supplementary Tables 6–7). The most significant novel locus, rs687339 (*P*_PLEIO_ = 5·85 × 10^–10^ for TotFA-OA), interacts with *MSL2* and *PCCB* through three-dimensional chromatin looping. *MSL2* plays a role in regulating biallelic gene expression in mammals^(38)^, while *PCCB* is closely linked to both MUFA and PUFA (online Supplementary Table 6)^(18,34,39)^.

### Colocalisation analysis

To prioritise candidate causal variants, we calculated the posterior probabilities for five hypotheses of all loci identified by PLEIO using Coloc. Among the sixty-eight significant pleiotropic loci, twenty-eight had a PP4 greater than 0·50, suggesting that these associations may be driven by the same underlying causal variant. These twenty-eight loci included seven loci shared with TotFA, nine with SFA, five with MUFA and seven with PUFA (online Supplementary Table 8 and 9). Notably, rs429358 and rs7412 were potential causal variants that regulated all four fatty acids and OA (online Supplementary Figure 7).

Of the nine novel pleiotropic loci, three demonstrated colocalisation: rs687339 for TotFA-OA (PP4 = 0·57), rs687339 for SFA-OA (PP4 = 0·54) and rs193084249 for MUFA-OA (PP4 = 0·58) (online Supplementary Table 6).

### Gene-based analysis

To further investigate the genetic mechanisms linking fatty acids and OA, we utilised MAGMA to identify candidate genes associated with each trait. An independent set of significant associated genes (*P* < 1 × 10^–3^) was identified for each trait, including 563 genes for TotFA, 449 for SFA, 546 for MUFA, 613 for PUFA and 378 for OA. Among these identified genes, PUFA shared the largest number of genes with OA (*n* 45, of which 25 were novel), followed by TotFA (*n* 30, of which 17 were novel) and SFA (*n* 23, of which 15 were novel), while MUFA shared the fewest genes (*n* 17, of which 16 were novel) (online Supplementary Tables 10–13). Notably, among the novel shared genes, *NUDCD3*, *NIP7*, *CYP2W1* and *KDF1* were found to be common across all four fatty acids. *KDF1*, located near the colocalised novel pleiotropic locus rs193084249, was a recently identified gene related to tooth development^(40)^.

### Enrichment analysis

**Figure 5.**
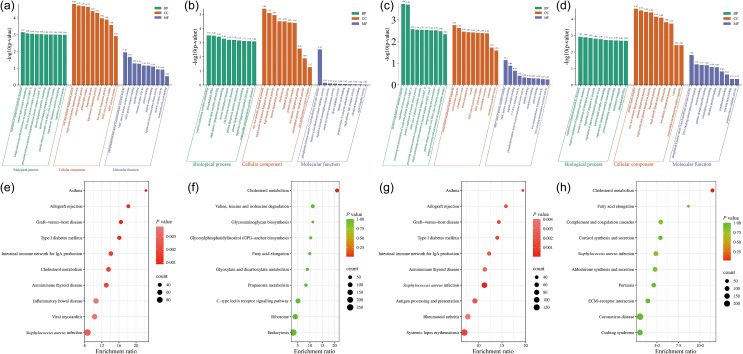
Enrichment analysis between fatty acids and OA. (a) GO function analysis histogram for TotFA and OA. The GO analysis categorizes gene functions into three components: biological process (BP), cellular component (CC) and molecular function (MF). BP is marked by green; CC is marked by orange and MF is marked by purple. (b) GO function analysis histogram for SFA and OA. (c) GO function analysis histogram for MUFA and OA. (d) GO function analysis histogram for PUFA and OA. (e) Dot plot of the KEGG pathway enrichment analysis between TotFA and OA. The horizontal axis represents the gene ratio, while the vertical axis represents the enriched pathway name. The color scale indicates different thresholds of the *P* value, and the size of the dot indicates the number of genes corresponding to each pathway. (f) Dot plot of the KEGG pathway enrichment analysis between SFA and OA. (g) Dot plot of the KEGG pathway enrichment analysis between MUFA and OA. (h) Dot plot of the KEGG pathway enrichment analysis between PUFA and OA. GO, Gene Ontology; KEGG, Kyoto Encyclopedia of Genes and Genomes; OA, osteoarthritis.

We performed enrichment analysis to elucidate the potential biological mechanisms of the shared genes identified by PLEIO and MAGMA. Distinct enrichment patterns across TotFA, SFA, MUFA and PUFA were exhibited by GO and KEGG analyses (Figure 5). GO analysis indicated that genes associated with TotFA-OA and MUFA-OA were enriched in immune-related pathways, including antigen processing and presentation of peptide antigens via MHC class II and positive regulation of immune response (Figure 5(a) and (c)). In contrast, for SFA and PUFA, enrichment was found in lipoprotein-related pathways, such as very-low-density lipoprotein particles and plasma lipoprotein particles (Figure 5(b) and (d)). KEGG analysis revealed that genes associated with TotFA and MUFA were enriched in immune and inflammatory response pathways, including allograft rejection and staphylococcus aureus infection (Figure 5(e) and (g)), while in the case of SFA and PUFA, enrichment was only identified in the cholesterol metabolism pathway (Figure 5(f) and (h)).

### Mendelian randomisation analysis

**Figure 6.**
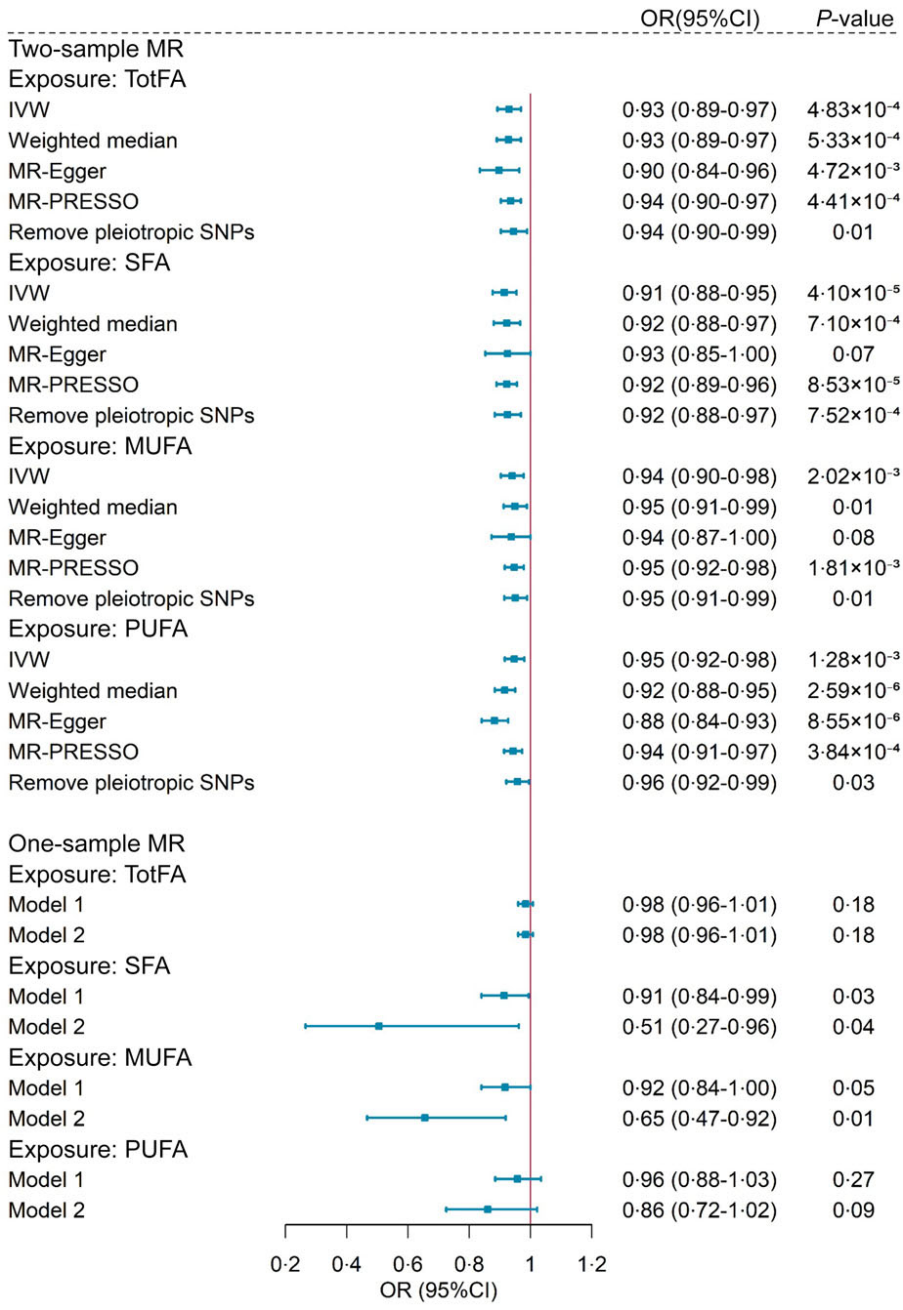
Figure 6 long description. Two-sample and one-sample MR analyses between fatty acid and OA. Blue boxes denote point estimates of the causal effects and error bars denote 95 % CI. MR, Mendelian randomization; OA, osteoarthritis.

In two-sample MR, we observed that each one standard deviation increase in genetically predicted TotFA (OR 0·93; 95 % CI 0·89, 0·97), SFA (OR 0·91; 95 % CI 0·88, 0·95), MUFA (OR 0·94; 95 % CI 0·90, 0·98) and PUFA (OR 0·95; 95 % CI 0·92, 0·98) was all significantly associated with a reduced risk of OA (all *P* < 0·05 and FDR-corrected *P* < 0·05, Figure 6 and online Supplementary Table 18). The directions of estimates from sensitivity analyses aligned with those of the inverse-variance weighted method. Estimates remained stable after excluding specific pleiotropic variants and outliers (online Supplementary Figure 2). Leave-one-out and single-SNP plots are provided in online Supplementary Figure 3 and 4, and statistical tests for heterogeneity and pleiotropy are reported in online Supplementary Table 19. However, in multivariable MR analyses adjusting for the other fatty acid subtypes, higher genetically predicted SFA levels were associated with an increased risk of OA (OR 1·30; 95 % CI 1·03, 1·66), whereas the inverse associations for MUFA (OR 0·89; 95 % CI 0·79, 0·99) and PUFA (OR 0·82; 95 % CI 0·72, 0·94) remained consistent and robust (online Supplementary Figure 5). Reverse MR analysis indicated no evidence of causality from OA to fatty acids (online Supplementary Figure 6, Supplementary Tables 20–21).

The one-sample linear MR demonstrated significant causal associations between specific fatty acids and OA according to basic model. Higher levels of SFA (OR 0·91; 95 % CI 0·84, 0·99) and MUFA (OR 0·92; 95 % CI 0·84, 1·00) were linked to reduced risk of OA. In the fully adjusted model, the effects stabilised at 0·51 (95 % CI 0·27, 0·96) for SFA and 0·65 (95 % CI 0·47, 0·92) for MUFA (Figure 6). Unfortunately, none of the estimates survived FDR correction and should therefore be regarded as supportive rather than definitive (online Supplementary Table 18). No evidence of non-linearity was found within the limits of statistical power (online Supplementary Figure 7 and 8, Supplementary Table 22).

Collectively, the FDR-corrected significant associations observed in multivariable two-sample MR, complemented by the directional consistency provided in one-sample MR, identified MUFA and PUFA as credible protective factors, whereas the evidence for SFA was less consistent.

## Discussion

Our study represents the first comprehensive investigation on the relationship of circulating fatty acids and OA through both observational and genetic analyses, leveraging large-scale individual-level data from UKB and the hitherto largest GWAS summary statistics. Observational analysis identified SFA as a risk factor, while MUFA and PUFA as protective factors for OA. MR analysis further confirmed the protective role of MUFA and PUFA using genetically predicted level of exposure. Additional genetic evidence supported the phenotypic associations through localised genomic correlations, common pleiotropic loci and biological pathways. These findings collectively highlight a phenotypic relationship as well as a shared genetic basis underlying circulating fatty acids and OA, particularly underscoring both MUFA and PUFA as key modifiable factors for OA prevention.

Epidemiological research on blood-based fatty acid biomarkers remains scarce, with most prior studies focusing on dietary intake. Compared with dietary assessments using FFQ that are prone to bias and measurement errors, circulating fatty acids are regarded as more reliable and precise biomarkers^(41)^. The pro-inflammatory and pro-apoptotic properties of SFA, demonstrated *i*n vitro through apoptosis induction in meniscus cells^(42,43)^, may partially explain its risk association with OA. Despite inconsistent findings in previous studies regarding the impact of MUFA and PUFA on OA risk^(9,10,44)^, our results offer robust evidence that elevated levels of these fatty acids are protective against OA. This aligns with recent research underscoring their roles in modulating inflammation^(45,46)^. The underlying mechanisms of MUFA’s protective effects are not fully elucidated; however, proposed pathways include modulation of the SIRT1/FOXO1 signaLling axis and the regulation of apoptosis^(47)^. For PUFA, the benefit is largely attributed to *n*-3 fatty acids such as EPA and DHA, which serve as precursors for pro-resolving mediators^(48)^. The role of *n*-6 PUFA is more complex. While they are precursors to pro-inflammatory eicosanoids, their overall impact in humans is nuanced, with some studies suggesting anti-inflammatory properties (online Supplementary Figure 9)^(49)^. The observed protective association for total PUFA likely arises from the combined and potentially balancing effects of these two fatty acid families.

Although the genome-wide genetic correlation between fatty acids and OA appeared minimal, we identified significant local correlations in three specific genomic regions. Notably, the 1p31·3 and 17q24·2 regions were consistently found for each of the four fatty acids and OA. Importantly, region 1p31·3 has been repeatedly implicated in bone metabolism and mineralisation, encompassing key genes that encode Wnt ligand secretion mediators, thereby modulating downstream Wnt/*β*-catenin signalling pathways^(50)^. This pathway plays a critical role in skeletal development and metabolism by regulating bone formation and resorption^(50)^.

The shared genetic basis may arise from pleiotropy, causality or an interplay of both. Through cross-trait meta-analysis, our study revealed pleiotropic characteristics, identifying sixty-eight significant pleiotropic loci of which nine were novel. We focused particularly on two colocalised novel loci, rs687339 and rs193084249. SNP rs687339 was identified as a novel shared SNP between TotFA and OA and was further validated in the context of SFA. This variant interacts with *MSL2* and *PCCB* genes via three-dimensional chromatin looping. The *MSL2* gene, part of the *MSL* complex, is involved in maintaining chromosomal integrity and stability^(51)^ and regulates chromatin remodelling, affecting fatty acid oxidation^(52)^ and catabolic activities in chondrocytes^(53)^. Additionally, the *PCCB* gene encodes a subunit of propionyl-CoA carboxylase, which plays a crucial role in the catabolism of certain amino acids and odd-chain fatty acids^(54)^. The *KDF1* gene, located near SNP rs193084249, was recognised in gene-based analysis as a shared gene between the four fatty acids and OA. *KDF1* is a key regulator of epidermal proliferation and differentiation^(55)^, and recent studies have linked it to tooth development^(40)^. Although current evidence linking *KDF1* to fatty acids or OA remains limited, significant interactions between *KDF1* and IKK*α* have been observed. IKK*α* is not only associated with PUFA^(56)^ but also functions as an essential kinase in the activation of NF-κB transcription factors, which are pivotal in cell differentiation and inflammation regulation^(57)^. This interaction hints at a plausible pathway through which *KDF1* may influence both fatty acids and OA.

Further enrichment analysis of pleiotropic genes showed significant involvement in lipoprotein metabolism, immune response and inflammation regulation pathways. Fatty acids usually serve as a major energy source for macrophages, supporting immune responses^(58)^. The activation of innate immunity can trigger inflammatory signalling, leading to immune cell infiltration in synovial tissue^(59)^. Additionally, distinct fatty acids exhibit differential pro- or anti-inflammatory effects^(60)^. These enriched pathways provide biological context for the observed genetic overlap.

Our MR analyses advance existing research in several key aspects: (i) leveraging the latest and largest GWAS of fatty acids, which provides more representative samples and robust genetic instruments; (ii) utilising individual-level data from the UKB, allowing for comprehensive adjustment for potential confounders; and (iii) incorporating non-linear effects, thereby addressing a critical gap in previous research. This study revealed a discrepancy between MR and observational studies regarding SFA. Multivariable MR resolved this paradox by showing that, after adjusting for MUFA and PUFA, SFA exhibits a direct harmful effect on OA risk. Also, this discrepancy can be understood in light of fatty acid biology. Circulating SFA levels are influenced not only by diet but also substantially by endogenous de novo lipogenesis, a metabolic process that concurrently affects multiple lipid fractions^(61)^. Thus, the MR estimate (influenced by intertwined metabolic pathways) and the observational association (reflecting long-term dietary patterns) likely capture different aspects of this complex biology.

Our findings carry important clinical and public health implications in the areas of prevention, diagnosis and treatment. Current OA management primarily relies on symptomatic treatments, such as nonsteroidal anti-inflammatory drugs and analgesics, which are insufficient in addressing OA’s progressive and multifactorial pathology^(62)^. For individuals with a genetic predisposition (e.g. at APOE), our results suggest that optimising dietary intake of MUFA and PUFA may offer a targeted, safe and preventive approach to reduce OA risk. Furthermore, the accessibility of circulating fatty acid measurement and their close biological link to OA position them as promising candidates for early diagnostic biomarkers. Finally, while direct clinical application requires further validation, the enriched pathways we identified provide a roadmap connecting modifiable risk factors to OA pathophysiology, revealing novel, biology-driven targets for developing disease-modifying therapies.

This study has several limitations. First, our findings, confined to populations of European ancestry, restrict the generalisability of results to other ethnic groups. Second, the GWAS of fatty acids and OA included individuals from the Rotterdam Studies, representing a sample overlap of 2·86 %. Following the method of Burgess *et al.* (https://sb452.shinyapps.io/overlap), we found the bias resulting from sample overlap was approximately 0·001, with an expected type I error rate of 0·05, indicating a negligible impact^(63)^ (online Supplementary Figure 10). Third, we did not formally test whether the observed effects are mediated by metabolic or inflammatory factors, as it would require additional robust genetic instruments and complex multivariable models. Fourth, one-sample MR analyses were constrained by sample size. This limitation resulted in more modest statistical precision for linear estimates and, when compounded by stratification in our exploratory non-linear analysis, reduced power to detect realistic dose-response patterns. Moreover, circulating fatty acid levels are influenced by a combination of exogenous and endogenous sources^(64)^, and although biomarkers partially reflect metabolic processes, observational estimates represent the net effect of these complex interactions. Finally, our study broadly classified fatty acids into four conventional categories based on their degree of saturation, lacking a more detailed classification; for instance, PUFA could be subdivided into *n*-3, *n*-6 and *n*-9 groups. This highlights the urgent need for a more detailed classification to better understand the nuanced relationships between specific fatty acids and OA.

### Conclusion

In conclusion, our study integrates observational and genetic analyses to elucidate the phenotypic association and genetic architecture linking circulating fatty acids with OA. We identified numerous pleiotropic loci and genes shared among TotFA, SFA, MUFA, PUFA and OA, as well as suggested a protective causal relationship of MUFA and PUFA on OA. These findings further substantiate the presence of shared biological processes underlying circulating fatty acids and OA, providing novel insights for disease prevention and intervention.

## Supporting information

Zhou et al. supplementary material Zhou et al. supplementary material

## Supplementary material

For supplementary material/s referred to in this article, please visit https://doi.org/10.1017/S0007114526106291.

## Figure 1 Long description

The flowchart outlines the study design for examining the association between fatty acids and osteoarthritis (OA) in European ancestry individuals. The process begins with the exposure to different types of fatty acids: total fatty acids (TotFA), saturated fatty acids (SFA), monounsaturated fatty acids (MUFA), and polyunsaturated fatty acids (PUFA). The study involves phenotypic association and genetic architecture analysis. Initially, 502,180 participants in the UK Biobank are considered. Non-white individuals (27,896) are excluded, leaving 474,284 white participants. Individuals with missing baseline measurements (214,908) are then excluded, resulting in 259,376 participants with baseline fatty acid data. Participants with diagnosed or self-reported OA at baseline (34,028) are excluded, retaining 225,348 participants. Outliers are excluded using the interquartile range (IQR) method. The retained participants undergo analysis using the Cox proportional hazards regression model and restricted cubic spline. On the genetic architecture side, genome-wide and local genetic correlation analyses are performed. Cross-trait meta-analysis, MAGMA analysis, two-sample Mendelian randomization (MR), one-sample MR, colocalization analysis, and enrichment analysis are conducted to investigate the genetic correlations and associations. The outcome of the study is the understanding of the relationship between fatty acids and OA.

Navigate back to Figure 1.

## Figure 4 Long description

Panel A: Circular Manhattan plot between total fatty acids (TotFA) and osteoarthritis (OA). The outermost circle shows the cross-trait meta-analysis results; inner circles show GWAS results for TotFA and OA, respectively. Light blue indicates genome-wide significant variants; dark blue indicates non-significant variants. SNPs are divided into four different categories according to their single-trait and cross-trait characteristics: single-trait-driven shared SNP (brown), LD-tagged shared SNP (purple), and novel shared SNP (red). Corresponding RS IDs are listed. Panel B: Circular Manhattan plot between saturated fatty acids (SFA) and OA. Panel C: Circular Manhattan plot between monounsaturated fatty acids (MUFA) and OA. Panel D: Circular Manhattan plot between polyunsaturated fatty acids (PUFA) and OA.

Navigate back to Figure 4.

## Figure 6 Long description

The table presents the results of two-sample and one-sample Mendelian randomization analyses examining the causal effects of various fatty acid exposures on osteoarthritis. It includes odds ratios (OR) with 95% confidence intervals (CI) and p-values for different methods and exposures. The table is divided into sections for two-sample MR and one-sample MR, with specific exposures listed under each section. For two-sample MR, exposures include TotFA, SFA, MUFA, and PUFA, analyzed using methods like IVW, Weighted median, MR-Egger, MR-PRESSO, and removal of pleiotropic SNPs. For one-sample MR, exposures include TotFA, SFA, MUFA, and PUFA, analyzed using Model 1 and Model 2. Each row provides the OR (95% CI) and p-value for the respective method and exposure. Notable trends include significant p-values and varying ORs across different methods and exposures, indicating the potential impact of fatty acids on osteoarthritis.

Navigate back to Figure 6.

## Table 1 Long description

A table comparing cases per person-years and hazard ratios for different fatty acids. The table has 4 rows and 6 columns. Column headers are n, Cases/Person-years, Model 1 HR, Model 1 95 % CI, Model 2 HR, Model 2 95 % CI. Row labels are TotFA, SFA, MUFA, PUFA. Row 1: TotFA, 211 161, 33 872/2 597 849, 1-00, 1-00, 1-01, 1-00, 1-01. Row 2: SFA, 209 999, 33 606/2 584 079, 1-01, 1-00, 1-02, 1-05, 1-03, 1-07. Row 3: MUFA, 209 548, 33 553/2 578 721, 1-01, 1-00, 1-02, 0-94, 0-90, 0-98. Row 4: PUFA, 212 267, 34 142/2 610 743, 0-99, 0-97, 1-00, 0-95, 0-93, 0-97.

Navigate back to Table 1.
