# Supplementary material for: Circulating fatty acids and osteoarthritis: evidence from observational and genetic analyses
Source: Br J Nutr. 2026 Feb 16;135(11):1247–57. doi: 10.1017/S0007114526106291 (PMC13423523; doi:10.1017/S0007114526106291)
Supplement: Zhou et al. supplementary material [file S0007114526106291sup001.pdf]

Table	Title
S1	Supplementary Table 1. Characteristics of datasets included in the study.
S2	Supplementary Table 2. Baseline Characteristics of UK Biobank participants grouped by TotFA.
S3	Supplementary Table 3. Baseline Characteristics of UK Biobank participants grouped by SFA.
S4	Supplementary Table 4. Baseline Characteristics of UK Biobank participants grouped by MUFA.
S5	Supplementary Table 5. Baseline Characteristics of UK Biobank participants grouped by PUFA.
S6	Supplementary Table 6. Novel pleiotropic loci between fatty acids and OA identified by PLEIO.
S7	Supplementary Table 7. Significant pleiotropic loci between fatty acids and OA identified by PLEIO.
S8	Supplementary Table 8. Results of colocalization analysis of pleiotropic loci between fatty acid and OA.
S9	Supplementary Table 9. Robustness of colocalization results.
S10	Supplementary Table 10. Genome-wide gene-based association study was performed in MAGMA based on the GWAS meta-analysis of TotFA and
S11	Supplementary Table 11. Genome-wide gene-based association study was performed in MAGMA based on the GWAS meta-analysis of SFA and O
S12	Supplementary Table 12. Genome-wide gene-based association study was performed in MAGMA based on the GWAS meta-analysis of MUFA and
S13	Supplementary Table 13. Genome-wide gene-based association study was performed in MAGMA based on the GWAS meta-analysis of PUFA and
S14	Supplementary Table 14. Details of instrument variables for TotFA.
S15	Supplementary Table 15. Details of instrument variables for SFA.
S16	Supplementary Table 16. Details of instrument variables for MUFA.
S17	Supplementary Table 17. Details of instrument variables for PUFA.
S18	Supplementary Table 18. False discovery rate (FDR)-adjusted *P*-values across MR analyses.
S19	Supplementary Table 19. Two-sample MR sensitivity analyses of fatty acids on OA.
S20	Supplementary Table 20. Steiger directionality test results for two-sample MR analyses.
S21	Supplementary Table 21. Summary of two-sample MR and sensitivity analyses.
S22	Supplementary Table 22. Instrument strength, statistical power and heterogeneity in nonlinear MR analyses.

Supplementary Table 1. Characteristics of datasets included in the study.

Phenotype	Data source	N	Reference genome	Population
TotFA	Karjalainen MK, Karthikeyan S, Oliver-Williams C, Sliz E, Allara E, Fung WT, Surendran P, Zhang W, Jousilahti P, Kristiansson K, Salomaa V, Goodwin M, Hughes DA, Boehnke M, Fernandes Silva L, Yin X, Mahajan A, Neville MJ, van Zuydam NR, de Mutsert R, Li-Gao R, Mook-Kanamori DO, Demirkan A, Liu J, Noordam R, Trompet S, Chen Z, Kartsonaki C, Li L, Lin K, Hagenbeek FA, Hottenga JJ, Pool R, Ikram MA, van Meurs J, Haller T, Milaneschi Y, Kähönen M, Mishra PP, Joshi PK, Macdonald-Dunlop E, Mangino M, Zierer J, Acar IE, Hoyng CB, Lechanteur YTE, Franke L, Kurilshikov A, Zhernakova A, Beekman M, van den Akker EB, Kolcic I, Polasek O, Rudan I, Gieger C, Waldenberger M, Asselbergs FW; China Kadoorie Biobank Collaborative Group; Estonian Biobank Research Team; FinnGen; Hayward C, Fu J, den Hollander AI, Menni C, Spector TD, Wilson JF, Lehtimäki T, Raitakari OT, Penninx BWJH, Esko T, Walters RG, Jukema JW, Sattar N, Ghanbari M, Willems van Dijk K, Karpe F, McCarthy MI, Laakso M, Järvelin MR, Timpson NJ, Perola M, Kooner JS, Chambers JC, van Duijn C, Slagboom PE, Boomsma DI, Danesh J, Ala-Korpela M, Butterworth AS, Kettunen J. Genome-wide characterization of circulating metabolic biomarkers. [illegible]	136016	GRCH37	European
SFA		136,016	GRCH37	European
MUFA		136,016	GRCH37	European
PUFA		136,016	GRCH37	European
OA	Boer CG, Hatzikotoulas K, Southam L, Stefánsdóttir L, Zhang Y, Coutinho de Almeida R, Wu TT, Zheng J, Hartley A, Teder-Laving M, Skogholt AH, Terao C, Zengini E, Alexiadis G, Barysenka A, Bjornsdottir G, Gabrielsen ME, Gilly A, Ingvarsson T, Johnsen MB, Jonsson H, Kloppenburg M, Luetge A, Lund SH, Mägi R, Mangino M, Nelissen RRGHH, Shivakumar M, Steinberg J, Takuwa H, Thomas LF, Tuerlings M; arcOGEN Consortium; HUNT All-In Pain; ARGO Consortium; Regeneron Genetics Center; Babis GC, Cheung JPY, Kang JH, Kraft P, Lietman SA, Samartzis D, Slagboom PE, Stefansson K, Thorsteinsdottir U, Tobias JH, Uitterlinden AG, Winsvold B, Zwart JA, Davey Smith G, Sham PC, Thorleifsson G, Gaunt TR, Morris AP, Valdes AM, Tsezou A, Cheah KSE, Ikegawa S, Hveem K, Esko T, Wilkinson JM, Meulenbelt I, Lee MTM, van Meurs JBJ, Styrkársdóttir U, Zeggini E. Deciphering osteoarthritis genetics across 826,690 individuals from 9 populations. Cell. 2021 Sep 2;184(18):4784-4818.e17.	826,690	GRCH37	European

TotFA, total fatty acid; SFA, saturated fatty acid; MUFA, monounsaturated fatty acid; PUFA, polyunsaturated fatty acid; OA, osteoarthritis; N, sample size.

Supplementary Table 2. Baseline Characteristics of UK Biobank participants grouped by TotFA.

Characteristics	TotFA				
	All	Q1 (5.76-10.35)	Q2 (10.35-11.73)	Q3 (11.73-13.31)	Q4 (13.31-18.10)
**Total (N)**	211,161	52,793	52,807	52,795	52,766
**Age at recruitment (years), mean ± SD**	56.22 ± 8.09	54.64 ± 8.71	55.99 ± 8.11	56.99 ± 7.72	57.25 ± 7.49
**Body mass index (BMI, kg/m^2), mean ± SD**	26.79 ± 4.00	26.02 ± 3.99	26.39 ± 3.94	26.96 ± 3.97	27.79 ± 3.87
**Townsend deprivation index at recruitment**	-1.66 ± 2.81	-1.60 ± 2.83	-1.70 ± 2.80	-1.69 ± 2.80	-1.63 ± 2.81
**Sex**					
Female	111,735 (52.91%)	24,721 (46.83%)	28,567 (54.10%)	29,486 (55.85%)	28,961 (54.89%)
Male	99,426 (47.09%)	28,072 (53.17%)	24,240 (45.90%)	23,309 (44.15%)	23,805 (45.11%)
**Assessment center**					
England	191,911 (90.88%)	47,734 (90.42%)	47,932 (90.77%)	48,096 (91.10%)	48,149 (91.25%)
Scotland	12,474 (5.91%)	3,299 (6.25%)	3,236 (6.13%)	3,004 (5.69%)	2,935 (5.56%)
Wales	6,776 (3.21%)	1,760 (3.33%)	1,639 (3.10%)	1,695 (3.21%)	1,682 (3.19%)
**Genotype measurement batch**					
Axiom	188,517 (89.28%)	47,329 (89.65%)	47,395 (89.75%)	46,983 (88.99%)	46,810 (88.71%)
BiLEVE	22,644 (10.72%)	5,464 (10.35%)	5,412 (10.25%)	5,812 (11.01%)	5,956 (11.29%)
**Education**					
College or University degree	69,867 (33.09%)	19,361 (36.67%)	18,040 (34.16%)	16,846 (31.91%)	15,620 (29.60%)
Non-degree qualifications	141,294 (66.91%)	33,432 (63.33%)	34,767 (65.84%)	35,949 (68.09%)	37,146 (70.40%)
**Smoking status**					
Never	116,590 (55.21%)	30,415 (57.61%)	29,775 (56.38%)	28,920 (54.78%)	27,480 (52.08%)
Previous	72,943 (34.54%)	17,318 (32.80%)	17,976 (34.04%)	18,520 (35.08%)	19,129 (36.25%)
Current	21,628 (10.24%)	5,060 (9.58%)	5,056 (9.57%)	5,355 (10.14%)	6,157 (11.67%)
**Alcohol drinker status**					
Never	62,111 (18.32%)	1,601 (3.03%)	1,600 (3.03%)	1,540 (2.92%)	1,525 (2.89%)
Previous	139,187 (41.06%)	2,071 (3.92%)	1,629 (3.08%)	1,463 (2.77%)	1,414 (2.68%)
Current	137,720 (40.62%)	49,121 (93.04%)	49,578 (93.89%)	49,792 (94.31%)	49,827 (94.43%)

Baseline characteristics of UK Biobank were presented as mean ± standard deviation for continuous variables, and as frequencies for categorical
TotFA, total fatty acid; SFA, saturated fatty acid; MUFA, monounsaturated fatty acid; PUFA, polyunsaturated fatty acid.

Supplementary Table 3. Baseline Characteristics of UK Biobank participants grouped by SFA.

Characteristics	SFA				
	All	Q1 (1.68-3.45)	Q2 (3.45-3.96)	Q3 (3.96-4.57)	Q4 (4.57-6.42)
**Total (N)**	209,999	52,511	52,494	52,499	52,495
**Age at recruitment (years), mean ± SD**	56.22 ± 8.09	54.64 ± 8.68	55.91 ± 8.12	56.99 ± 7.73	57.33 ± 7.51
**Body mass index (BMI, kg/m^2), mean ± SD**	26.77 ± 3.99	25.96 ± 3.93	26.34 ± 3.92	26.92 ± 3.96	27.87 ± 3.90
**Townsend deprivation index at recruitment**	-1.66 ± 2.81	-1.60 ± 2.84	-1.71 ± 2.79	-1.70 ± 2.80	-1.62 ± 2.82
**Sex**					
Female	111,408 (53.05%)	24,554 (46.76%)	29,020 (55.28%)	29,821 (56.80%)	28,013 (53.36%)
Male	98,591 (46.95%)	27,957 (53.24%)	23,474 (44.72%)	22,678 (43.20%)	24,482 (46.64%)
**Assessment center**					
England	190,847 (90.88%)	47,471 (90.40%)	47,690 (90.85%)	47,863 (91.17%)	47,823 (91.10%)
Scotland	12,424 (5.92%)	3,246 (6.18%)	3,142 (5.99%)	3,060 (5.83%)	2,976 (5.67%)
Wales	6,728 (3.20%)	1,794 (3.42%)	1,662 (3.17%)	1,576 (3.00%)	1,696 (3.23%)
**Genotype measurement batch**					
Axiom	187,502 (89.29%)	47,160 (89.81%)	47,143 (89.81%)	46,802 (89.15%)	46,397 (88.38%)
BiLEVE	22,497 (10.71%)	5,351 (10.19%)	5,351 (10.19%)	5,697 (10.85%)	6,098 (11.62%)
**Education**					
College or University degree	69,555 (33.12%)	19,473 (37.08%)	17,935 (34.17%)	16,677 (31.77%)	15,470 (29.47%)
Non-degree qualifications	140,444 (66.88%)	33,038 (62.92%)	34,559 (65.83%)	35,822 (68.23%)	37,025 (70.53%)
**Smoking status**					
Never	116,139 (55.30%)	30,833 (58.72%)	29,858 (56.88%)	28,644 (54.56%)	26,804 (51.06%)
Previous	72,427 (34.49%)	16,965 (32.31%)	17,680 (33.68%)	18,445 (35.13%)	19,337 (36.84%)
Current	21,433 (10.21%)	4,713 (8.98%)	4,956 (9.44%)	5,410 (10.30%)	6,354 (12.10%)
**Alcohol drinker status**					
Never	6,241 (2.97%)	1,649 (3.14%)	1,626 (3.10%)	1,546 (2.94%)	1,420 (2.71%)
Previous	6,552 (3.12%)	2,184 (4.16%)	1,603 (3.05%)	1,424 (2.71%)	1,341 (2.55%)
Current	197,206 (93.91%)	48,678 (92.70%)	49,265 (93.85%)	49,529 (94.34%)	49,734 (94.74%)

Baseline characteristics of UK Biobank were presented as mean ± standard deviation for continuous variables, and as frequencies for categorical

TotFA, total fatty acid; SFA, saturated fatty acid; MUFA, monounsaturated fatty acid; PUFA, polyunsaturated fatty acid.

Supplementary Table 4. Baseline Characteristics of UK Biobank participants grouped by MUFA.

Characteristics	MUFA				
	All	Q1 (0.82-2.29)	Q2 (2.29-2.72)	Q3 (2.72-3.26)	Q4 (3.26-4.87)
**Total (N)**	209,548	52,399	52,376	52,386	52,387
**Age at recruitment (years), mean ± SD**	56.23 ± 8.09	54.56 ± 8.56)	56.26 ± 8.05	57.04 ± 7.79	57.04 ± 7.66
**Body mass index (BMI, kg/m^2), mean ± SD**	26.76 ± 3.99	25.37 ± 3.75)	26.23 ± 3.84	27.18 ± 3.93	28.27 ± 3.83
**Townsend deprivation index at recruitment**	-1.66 ± 2.81	-1.72 ± 2.78)	-1.72 ± 2.77	-1.66 ± 2.82	-1.54 ± 2.86
**Sex**					
Female	111,523 (53.22%)	28,608 (54.60%)	29,644 (56.60%)	28,383 (54.18%)	24,888 (47.51%)
Male	98,025 (46.78%)	23,791 (45.40%)	22,732 (43.40%)	24,003 (45.82%)	27,499 (52.49%)
**Assessment center**					
England	190,474 (90.90%)	47,289 (90.25%)	47,705 (91.08%)	47,712 (91.08%)	47,768 (91.18%)
Scotland	12,381 (5.91%)	3,384 (6.46%)	3,069 (5.86%)	3,022 (5.77%)	2,906 (5.55%)
Wales	6,693 (3.19%)	1,726 (3.29%)	1,602 (3.06%)	1,652 (3.15%)	1,713 (3.27%)
**Genotype measurement batch**					
Axiom	187,167 (89.32%)	47,393 (90.45%)	47,045 (89.82%)	46,553 (88.87%)	46,176 (88.14%)
BiLEVE	22,381 (10.68%)	5,006 (9.55%)	5,331 (10.18%)	5,833 (11.13%)	6,211 (11.86%)
**Education**					
College or University degree	69,525 (33.18%)	20,504 (39.13%)	17,907 (34.19%)	16,200 (30.92%)	14,914 (28.47%)
Non-degree qualifications	140,023 (66.82%)	31,895 (60.87%)	34,469 (65.81%)	36,186 (69.08%)	37,473 (71.53%)
**Smoking status**					
Never	116,065 (55.39%)	31,259 (59.66%)	29,852 (57.00%)	28,514 (54.43%)	26,440 (50.47%)
Previous	72,298 (34.50%)	16,917 (32.28%)	17,738 (33.87%)	18,437 (35.19%)	19,206 (36.66%)
Current	21,185 (10.11%)	4,223 (8.06%)	4,786 (9.14%)	5,435 (10.37%)	6,741 (12.87%)
**Alcohol drinker status**					
Never	6,234 (2.97%)	1,473 (2.81%)	1,540 (2.94%)	1,631 (3.11%)	1,590 (3.04%)
Previous	6,519 (3.11%)	1,771 (3.38%)	1,578 (3.01%)	1,562 (2.98%)	1,608 (3.07%)
Current	196,795 (93.91%)	49,155 (93.81%)	49,258 (94.05%)	49,193 (93.90%)	49,189 (93.90%)

Baseline characteristics of UK Biobank were presented as mean ± standard deviation for continuous variables, and as frequencies for categorical

TotFA, total fatty acid; SFA, saturated fatty acid; MUFA, monounsaturated fatty acid; PUFA, polyunsaturated fatty acid.

Supplementary Table 5. Baseline Characteristics of UK Biobank participants grouped by PUFA.

Characteristics	PUFA				
	All	Q1 （2.90-4.49）	Q2 （4.49-4.98）	Q3 （4.98-5.51）	Q4 （5.51-7.12）
**Total (N)**	212,267	53,076	53,058	53,068	53,065
**Age at recruitment (years), mean ± SD**	56.20 ± 8.09	55.18 ± 8.77	55.65 ± 8.22	56.48 ± 7.78	57.51 ± 7.32
**Body mass index (BMI, kg/m^2), mean ± SD**	26.82 ± 4.01	26.82 ± 4.20	26.72 ± 4.02	26.78 ± 3.96	26.97 ± 3.84
**Townsend deprivation index at recruitment**	-1.65 ± 2.81	-1.48 ± 2.89	-1.64 ± 2.82	-1.74 ± 2.77	-1.75 ± 2.76
**Sex**					
Female	111,854 (52.69%)	21,094 (39.74%)	26,398 (49.75%)	30,138 (56.79%)	34,224 (64.49%)
Male	100,413 (47.31%)	31,982 (60.26%)	26,660 (50.25%)	22,930 (43.21%)	18,841 (35.51%)
**Assessment center**					
England	192,915 (90.88%)	47,977 (90.39%)	48,181 (90.81%)	48,283 (90.98%)	48,474 (91.35%)
Scotland	12,551 (5.91%)	3,336 (6.29%)	3,204 (6.04%)	3,041 (5.73%)	2,970 (5.60%)
Wales	6,801 (3.20%)	1,763 (3.32%)	1,673 (3.15%)	1,744 (3.29%)	1,621 (3.05%)
**Genotype measurement batch**					
Axiom	189,412 (89.23%)	47,001 (88.55%)	47,373 (89.29%)	47,480 (89.47%)	47,558 (89.62%)
BiLEVE	22,855 (10.77%)	6,075 (11.45%)	5,685 (10.71%)	5,588 (10.53%)	5,507 (10.38%)
**Education**					
College or University degree	70,029 (32.99%)	17,764 (33.47%)	17,867 (33.67%)	17,495 (32.97%)	16,903 (31.85%)
Non-degree qualifications	142,238 (67.01%)	35,312 (66.53%)	35,191 (66.33%)	35,573 (67.03%)	36,162 (68.15%)
**Smoking status**					
Never	116,824 (55.04%)	28,439 (53.58%)	29,409 (55.43%)	29,475 (55.54%)	29,501 (55.59%)
Previous	73,453 (34.60%)	18,202 (34.29%)	18,161 (34.23%)	18,351 (34.58%)	18,739 (35.31%)
Current	21,990 (10.36%)	6,435 (12.12%)	5,488 (10.34%)	5,242 (9.88%)	4,825 (9.09%)
**Alcohol drinker status**					
Never	6,283 (2.96%)	1,651 (3.11%)	1,550 (2.92%)	1,503 (2.83%)	1,579 (2.98%)
Previous	6,584 (3.10%)	2,161 (4.07%)	1,620 (3.05%)	1,454 (2.74%)	1,349 (2.54%)
Current	199,400 (93.94%)	49,264 (92.82%)	49,888 (94.03%)	50,111 (94.43%)	50,137 (94.48%)

Baseline characteristics of UK Biobank were presented as mean ± standard deviation for continuous variables, and as frequencies for categorical
TotFA, total fatty acid; SFA, saturated fatty acid; MUFA, monounsaturated fatty acid; PUFA, polyunsaturated fatty acid.

Supplementary Table 6. Novel pleiotropic loci between fatty acids and OA identified by PLEIO.

FA	SNP	Chr:Position	*P*-value			Ranges	Linear closest	Interacting genes	PPH4
			FA	OA	PLEIO				
TotFA	rs687339	chr3:135798658	5.73E-07	1.32E-05	5.85E-10	MSL2,PCCB,PPP2R3A,STAG1	—	MSL2,PCCB	0.572
	rs7841093	chr8:10584212	9.79E-07	2.76E-04	1.73E-08	MIR1322,PINX1,SOX7	PINX1, SOX7	C8orf74,MIR1322,PINX1,PRSS55,RP1L1,SOX7	0.004
SFA	rs687339	chr3:135798658	1.36E-06	1.32E-05	1.37E-09	MSL2,PCCB,PPP2R3A,STAG1	—	MSL2,PCCB	0.538
MUFA	rs193084249	chr1:26987646	2.08E-07	4.34E-04	4.96E-09	ARID1A,GPATCH3,GPN2,KDF1,NR0B2,NUDC,PIGV,SFN,ZDHHC18	—	ARID1A,MIR1976,PIGV,RPS6KA1	0.582
	rs6913037	chr6:34988391	3.66E-07	9.97E-05	2.07E-09	ANKS1A,DEF6,PPARD,SCUBE3,TCP11,ZNF76	DEF6	DEF6,FANCE,PPARD,RPL10A,ZNF76	0.241
	rs7813718	chr8:9181764	3.06E-04	2.06E-06	2.35E-08	LINC00599,LOC157273,MIR597,MSRA,MIR124-1,TNKS	—	MIR597	0.216
	rs9302635	chr16:72078043	9.57E-07	7.99E-04	3.54E-08	DHX38,HP,HPR,PMFBP1,TXNL4B	DHX38,PMFBP1	DHX38,HP,HPR,PMFBP1,TXNL4B	0.019
	rs4252548	chr19:55879672	9.25E-05	5.95E-06	2.16E-08	IL11	IL11	BRSK1,COX6B2,FAM71E2,HSPBP1,IL11,ISOC2,PPP6R1,PTPRH,RPL28,SHISA7,SUV420H2,TMEM150B,TMEM190,TMEM238,TMEM86B,UBE2S,ZNF628	0.034
PUFA	rs210157	chr6:33341424	5.06E-05	9.15E-07	3.03E-09	CUTA,KIFC1,MIR5004,PHF1,SYNGAP1,ZBTB	—	—	0.071

SNP, single nucleotide polymorphism; Chr, chromosome; OA, osteoarthritis; PP4, posterior probability for H4 from colocalization analysis.
FA, fatty acid; TotFA, total fatty acid; SFA, saturated fatty acid; MUFA, monounsaturated fatty acid; PUFA, polyunsaturated fatty acid.

Supplementary Table 7. Significant pleiotropic loci between fatty acids and OA identified by PLEIO.

FA	SNP	Chr:Position	*P*-value			Ranges	Linear closest	Interacting genes	PPH4
			FA	OA	PLEIO				
single-trait-driven shared SNP									
TotFA	rs79598313	chr1:26987646	1.20E-10	7.65E-04	8.74E-12	[ARID1A,FAM46B,GPATCH3,GPN2,KDF1,NR0B2,NUDC,PIGV,SFN,TRNP1,ZDHHC18]	C1orf172	C1orf172, FAM46B, GPATCH3, GPN2, NR0B2, NUDC, SFN, TRNP1	0.628
	rs11690912	chr2:20419112	1.31E-10	1.04E-05	3.78E-14	[PUM2,SDC1]	PUM2	PUM2, SDC1	0.004
	rs9394283	chr6:34988391	1.45E-12	5.46E-05	1.19E-15	[ANKS1A,DEF6,PPARD,SCUBE3,TCP11,ZNF76]	SCUBE3, RP3-	SCUBE3, TCP11, ZNF76	0.215
	rs2965169	chr19:45224305	1.21E-13	7.32E-04	1.45E-15	—	BCL3	BCAM,BCL3,CBLC,CEACAM16,CEACAM19,IGSF23,MIR4531,PVR,PVRL2	0.998
	rs429358	chr19:45387459	1.78E-123	6.08E-07	2.12E-163	[APOC1,APOE,PVRL2,TOMM40]	APOE, TOMM40	APOC1, APOC1P1, APOC2, APOC4, APOC4-APOC2, APOE, BCAM, CLASRP, CLPTM1, GEMIN7, PVRL2, RELB, [illegible]	0.998
	rs7412	chr19:45232161	7.89E-113	5.76E-05	1.09E-146	[APOC1,APOE,BCAM,BCL3,CBLC,MIR8085,PVRL2,TOMM40]	APOE	APOC1, APOC1P1, APOC2, APOC4, APOC4-APOC2, APOE, BCAM, CLASRP, CLPTM1, GEMIN7, PVRL2, RELB, [illegible]	0.998
	rs13107325	chr4:102702364	6.63E-05	3.25E-17	1.63E-22	[BANK1,SLC39A8]	SLC39A8	BANK1	0.939
	rs62033403	chr16:53797908	2.64E-04	2.94E-08	3.70E-10	[FTO]	FTO	FTO, RPGRIP1L	0.473
SFA	rs79598313	chr1:26987646	4.39E-10	7.65E-04	4.01E-11	[ARID1A,FAM46B,GPATCH3,GPN2,KDF1,NR0B2,NUDC,PIGV,SFN,TRNP1,ZDHHC18]	C1orf172	C1orf172, FAM46B, GPATCH3, GPN2, NR0B2, NUDC, SFN, TRNP1	0.627
	rs11690912	chr2:20437384	4.95E-09	1.04E-05	3.28E-12	[PUM2]	PUM2	PUM2, SDC1	0.004
	rs9394283	chr6:34988391	7.75E-13	5.46E-05	6.00E-16	[ANKS1A,DEF6,PPARD,SCUBE3,TCP11,ZNF76]	SCUBE3, RP3-	SCUBE3, TCP11, ZNF76	0.216
	rs115785198	chr19:45220896	3.70E-20	4.22E-04	3.40E-24	[BCL3]	BCL3	BCAM,BCL3,CBLC,CEACAM16,CEACAM19,IGSF23,MIR4531,PVR,PVRL2	0.998
	rs2965169	chr19:45242740	1.21E-09	7.32E-04	9.97E-11	—	BCL3	BCAM,BCL3,CBLC,CEACAM16,CEACAM19,IGSF23,MIR4531,PVR,PVRL2	0.998
	rs429358	chr19:45387459	4.82E-87	6.08E-07	1.53E-115	[APOC1,APOE,PVRL2,TOMM40]	APOE, TOMM40	APOC1, APOC1P1, APOC2, APOC4, APOC4-APOC2, APOE, BCAM, CLASRP, CLPTM1, GEMIN7, PVRL2, RELB, [illegible]	0.998
	rs7412	chr19:45232161	6.16E-84	5.76E-05	9.11E-109	[APOC1,APOE,BCAM,BCL3,CBLC,MIR8085,PVRL2,TOMM40]	APOE	APOC1, APOC1P1, APOC2, APOC4, APOC4-APOC2, APOE, BCAM, CLASRP, CLPTM1, GEMIN7, PVRL2, RELB, [illegible]	0.998
	rs13107325	chr4:102702364	8.28E-06	3.25E-17	1.11E-23	[BANK1,SLC39A8]	SLC39A8	BANK1	0.991
	rs476330	chr6:116314563	9.38E-04	4.51E-08	1.70E-09	[COL10A1,FRK,NT5DC1,TPI1P3]	NT5DC1	COL10A1,FRK,NT5DC1,TPI1P3,TSPYL4	0.008
	rs62033403	chr16:53797908	9.35E-05	2.94E-08	1.55E-10	[FTO]	FTO	FTO, RPGRIP1L	0.677
MUFA	rs11690912	chr2:20419112	9.52E-10	1.04E-05	2.55E-13	[PUM2,SDC1]	PUM2	PUM2, SDC1	0.004
	rs41288799	chr2:27155157	6.25E-10	1.12E-04	3.28E-12	[ABHD1,AGBL5,AGBL5-AS1,ATRAID,CAD,CGREF1,DNAJC5G,DPYSL5,EIF2B4,EMILIN1,GTF3C2,GTF3C2-AS1,KHK,MAPRE3,MPV17,OST4,PPM1G,PREB,PRR30,SLC5A6,SLC30A3,SNX17,TCF23,TMEM214,TRIM54,UCN,ZNF513]	PREB, ABHD1	ABHD1,AGBL5,ATRAID,C2orf53,CAD,CGREF1,DNAJC5G,EMILIN1,KHK,OST4,PREB,SLC30A3,SLC5A6,TCF23,TRIM54,UCN	0.003
	rs687339	chr3:135798658	9.12E-10	1.32E-05	3.52E-13	[MSL2,PCCB,PPP2R3A,STAG1]	—	MSL2,PCCB	0.631
	rs9469089	chr6:31800707	2.02E-11	2.50E-05	6.21E-15	[AGER,AGPAT1,ATF6B,C2,C4A,C4B,C4B_2,C6orf48,CFB,CYP21A1P,CYP21A2,DXO,EGFL8,EHMT2,FKBPL,GPSM3,LOC100507547,LOC102060414,MIR1236,MIR6721,MIR6833,NELFE,NEU1,NOTCH4,PBX2,PPT2,PPT2-EGFL8,PRRT1,RNF5,RNF5P1,SKIV2L,SLC44A4,SNORD48,SNORD52,STK19,TNXA,TNXB,ZBTB12]	AGPAT1, AGER, RNF5	AGER, AGPAT1, ATF6B, C2, C6orf47, CSNK2B, EGFL8, EHMT2, FKBPL, GPANK1, GPSM3, LOC100507547, NOTCH4, PBX2, PPT2, PPT2-EGFL8, PRRT1, RNF5, RNF5P1, TNXB, ZBTB12	0.003
	rs7841093	chr8:10584212	5.39E-09	2.76E-04	1.08E-10	[MIR1322,PINX1,SOX7]	PINX1, SOX7	C8orf74,MIR1322,PINX1,PRSS55,RP1L1,SOX7	0.007
	rs429358	chr19:45387459	2.45E-73	6.08E-07	1.48E-99	[APOC1,APOE,PVRL2,TOMM40]	APOE, TOMM40	APOC1, APOC1P1, APOC2, APOC4, APOC4-APOC2, APOE, BCAM, CLASRP, CLPTM1, GEMIN7, PVRL2, RELB, [illegible]	0.998
	rs58132661	chr19:45324138	1.21E-11	9.64E-04	3.39E-13	[BCAM,PVRL2]	BCAM	APOC1,APOC1P1,APOC2,APOC4,APOC4-APOC2,APOE,BCAM,BCL3,CBLC,CEACAM16,PVRL2,TOMM40	0.998
	rs7412	chr19:45232161	2.04E-11	5.76E-05	1.75E-14	[APOC1,APOE,BCAM,BCL3,CBLC,MIR8085,PVRL2,TOMM40]	APOE	APOC1, APOC1P1, APOC2, APOC4, APOC4-APOC2, APOE, BCAM, CLASRP, CLPTM1, GEMIN7, PVRL2, RELB, [illegible]	0.998
	rs79598313	chr1:26987646	2.80E-10	7.65E-04	4.48E-11	ARID1A,FAM46B,GPATCH3,GPN2,KDF1,NR0B2,NUDC,PIGV,SFN,TRNP1,ZDHHC18	C1orf172	C1orf172, FAM46B, GPATCH3, GPN2, NR0B2, NUDC, SFN, TRNP1	0.628
	rs11690912	chr2:20428737	1.54E-09	1.04E-05	1.57E-12	PUM2	PUM2	PUM2, SDC1	0.004
	rs112563428	chr6:34517229	6.63E-09	1.95E-04	2.04E-10	ANKS1A,C6orf106,SNRPC,SPDEF,TAF11,TCP11,UHRF1BP1	UHRF1BP1	ANKS1A, SNRPC, TAF11, UHRF1BP1	0.193
	rs3822856	chr6:116305637	5.97E-10	3.94E-04	4.40E-11	COL10A1,DSE,FRK,NT5DC1,TPI1P3,TSPYL1,TSPYL4	FRK	COL10A1, FRK, NT5DC1, TPI1P3	0.006
	rs9394283	chr6:34988391	6.47E-15	5.46E-05	3.15E-18	ANKS1A,DEF6,PPARD,SCUBE3,TCP11,ZNF76	SCUBE3, RP3-	SCUBE3, TCP11, ZNF76	0.220
	rs28399637	chr19:45324138	7.34E-27	9.31E-05	8.66E-33	BCAM	BCAM	APOC1, APOC1P1, APOC2, APOC4, APOC4-APOC2, APOE, BCAM, BCL3, CBLC, CEACAM16, PVRL2, TOMM40	0.862

PUFA	rs429358	chr19:45387459	1.19E-177	6.08E-07	4.45E-229	APOC1,APOE,PVRL2,TOMM40	APOE, TOMM40	APOC1, APOC1P1, APOC2, APOC4, APOC4-APOC2, APOE, BCAM, CLASRP, CLPTM1, GEMIN7, PVRL2, RELB, [illegible]	0.862
	rs7412	chr19:45232161	1.11E-308	5.76E-05	0	APOC1,APOC1P1,APOE,BCAM,BCL3,CBLC,MIR8085,PVRL2,TOMM40	APOE	APOC1, APOC1P1, APOC2, APOC4, APOC4-APOC2, APOE, BCAM, CLASRP, CLPTM1, GEMIN7, PVRL2, RELB, [illegible]	0.862
	rs13107325	chr4:102702364	3.23E-05	3.25E-17	9.41E-23	BANK1,SLC39A8	SLC39A8	BANK1	0.968
	rs62033403	chr16:53797908	6.83E-05	2.94E-08	1.28E-10	FTO	FTO	FTO, RPGRIP1L	0.714
	rs7248558	chr19:10748073	9.37E-04	1.57E-10	4.55E-12	DNM2,ILF3,ILF3-AS1,MIR638,QTRT1,SLC44A2	ILF3	AP1M2, ATG4D, CDKN2D, DNM2, ILF3, ILF3-AS1, KEAP1, KRI1, MIR1238, MIR638, QTRT1, S1PR5, SLC44A2	0.000
	rs6060660	chr20:34075532	1.58E-04	2.81E-08	2.50E-10	C20orf173,CEP250,CNBD2,CPNE1,ERGIC3,FER1L4,LINC00657,NFS1,PHF20,RBM12,RBM39,ROMO1,SCAND1,SPAG4	PHF20, RNU4-40P	CNBD2, CPNE1, PHF20, RBM39, SCAND1	0.003
LD-tagged shared SNP									
TotFA	rs113565903	chr1:26400806	3.18E-06	6.04E-05	1.17E-08	[CNKSR1,FAM110D,PDIK1L,ZNF593]	CATSPER4, CNKSR1	CATSPER4, CD52, CEP85, CNKSR1, EXTL1, FAM110D, LIN28A, PDIK1L, SH3BGRL3, SLC30A2, TRIM63, UBXN11, [illegible]	0.753
	rs10932008	chr2:203639395	7.61E-05	5.35E-08	2.22E-10	[ABI2,CARF,CYP20A1,ICA1L,NBEAL1,RAPH1,WDR12]	NBEAL1	CYP20A1	0.000
	rs1126513	chr6:33041064	4.27E-04	7.58E-08	1.44E-09	[COL11A2,HLA-DPA1,HLA-DPB1,HLA-DPB2]	HLA-DPB1,HLA-DPA1,RPL32P1,CR759904.1,AL805913.1	BRD2,COL11A2,HCG25,HLA-DMA,HLA-DMB,HLA-DOA,HLA-DPA1,HLA-DPB1,HLA-DPB2,HSD17B8,LOC100294145,MIR219-1,RING1,RXRB,SLC39A7	0.000
	rs1999659	chr6:116310243	3.80E-04	7.59E-08	1.27E-09	[COL10A1,FRK,NT5DC1,TPI1P3]	NT5DC1	COL10A1,FRK,NT5DC1,TPI1P3,TSPYL4	0.003
	rs9469089	chr6:32021130	1.23E-07	2.50E-05	2.67E-10	[AGER,AGPAT1,ATF6B,BTNL2,C6orf10,EGFL8,FKBPL,GPSM3,HCG23,LOC100507547,MIR6721,MIR6833,NOTCH4,PBX2,PPT2,PPT2-EGFL8,PRRT1,RNF5,RNF5P1,TNX[illegible]	AGPAT1, AGER, RNF5	AGER, AGPAT1, ATF6B, C2, C6orf47, CSNK2B, EGFL8, EHMT2, FKBPL, GPANK1, GPSM3, LOC100507547, NOTCH4, PBX2, PPT2, PPT2-EGFL8, PRRT1, RNF5, RNF5P1, TNXB, ZBTB12	0.002
	rs10251697	chr7:44347800	4.70E-06	8.68E-05	2.32E-08	[CAMK2B,NPC1L1,NUDCD3]	—	CAMK2B, NPC1L1, NUDCD3, YKT6	0.081
	rs62101766	chr18:47187455	1.95E-05	2.47E-05	2.53E-08	—	—	ACAA2, LIPG, RPL17, RPL17-C18orf32, SCARNA17, SNORD58A, SNORD58B	0.013
	rs12983316	chr19:10990329	1.50E-04	9.79E-07	6.83E-09	[C19orf52,CARM1,SMARCA4,YIP	SMARCA4	C19orf52, LDLR, SMARCA4, YIPF2	0.000
SFA	rs113565903	chr1:26400806	8.35E-06	6.04E-05	2.70E-08	[CNKSR1,FAM110D,PDIK1L,ZNF593]	CATSPER4, CNKSR1	CATSPER4, CD52, CEP85, CNKSR1, EXTL1, FAM110D, LIN28A, PDIK1L, SH3BGRL3, SLC30A2, TRIM63, UBXN11, [illegible]	0.753
	rs10932008	chr2:203639395	2.94E-04	5.35E-08	7.51E-10	[ABI2,CARF,CYP20A1,ICA1L,NBEAL1,RAPH1,WDR12]	NBEAL1	CYP20A1	0.001
	rs62101766	chr18:47187455	4.08E-06	2.47E-05	6.09E-09	—	—	ACAA2, LIPG, RPL17, RPL17-C18orf32, SCARNA17, SNORD58A, SNORD58B	0.013
MUFA	rs1563353	chr1:219622596	1.37E-04	2.17E-07	1.30E-09	—	—	—	0.197
	rs1126513	chr6:33041064	4.06E-05	7.58E-08	1.55E-10	[COL11A2,HLA-DPA1,HLA-DPB1,HLA-DPB2,HSD17B8,MIR219A1,RING1,RXRB,SLC39A7]	HLA-DPB1,HLA-DPA1,RPL32P1,CR759904.1,AL805913.1	BRD2,COL11A2,HCG25,HLA-DMA,HLA-DMB,HLA-DOA,HLA-DPA1,HLA-DPB1,HLA-DPB2,HSD17B8,LOC100294145,MIR219-1,RING1,RXRB,SLC39A7	0.000
	rs34421088	chr8:11392093	1.32E-05	3.15E-05	1.88E-08	[BLK,C8orf49,CTSB,DEFB134,DEFB135,DEFB136,FDFT1,GATA4,LINC00208,NEIL2]	GATA4	C8orf49,CTSB,FDFT1,GATA4,LINC00208, NEIL2	0.002
PUFA	rs113565903	chr1:26400806	7.54E-06	6.04E-05	3.36E-08	CNKSR1,FAM110D,PDIK1L,ZNF593	CATSPER4, CNKSR1	CATSPER4, CD52, CEP85, CNKSR1, EXTL1, FAM110D, LIN28A, PDIK1L, SH3BGRL3, SLC30A2, TRIM63, UBXN11, [illegible]	0.753
	rs10932008	chr2:203554324	4.42E-06	5.35E-08	1.87E-11	ABI2,CARF,CYP20A1,FAM117B,ICA1L,NBEAL1,RAPH1,WDR12	NBEAL1	CYP20A1	0.000
	rs9469089	chr6:31800707	1.19E-06	2.50E-05	2.68E-09	AGPAT1,ATF6B,C2,C4A,C4B,C4B_2,C6orf48,CFB,CYP21A1P,CYP21A2,DXO,EGFL8,EHMT2,FKBPL,LOC100507547,LOC102060414,MIR1236,MIR6721,NELFE,NEU1,PPT2,PPT2-EGFL8,PRRT1,RNF5,RNF5P1,SKIV2L,SLC44A4,SNORD48,SNORD52,STK19,TNXA,TNXB,ZBTB12	AGPAT1, AGER, RNF5	AGER, AGPAT1, ATF6B, C2, C6orf47, CSNK2B, EGFL8, EHMT2, FKBPL, GPANK1, GPSM3, LOC100507547, NOTCH4, PBX2, PPT2, PPT2-EGFL8, PRRT1, RNF5, RNF5P1, TNXB, ZBTB12	0.003
	rs10251697	chr7:44347800	2.04E-06	8.68E-05	1.45E-08	CAMK2B,NPC1L1,NUDCD3	—	CAMK2B, NPC1L1, NUDCD3, YKT6	0.035
	rs11774915	chr8:9083416	8.72E-06	6.37E-05	4.01E-08	LOC157273,MIR597,TNKS	RP11-115J16.1	LOC157273, MIR4660, PPP1R3B	0.000
	rs62101766	chr18:47183937	5.69E-06	2.47E-05	1.12E-08	—	—	ACAA2, LIPG, RPL17, RPL17-C18orf32, SCARNA17, SNORD58A, SNORD58B	0.013
	rs12983316	chr19:10990329	6.67E-07	9.79E-07	7.44E-11	C19orf52,CARM1,SMARCA4,YIPF	SMARCA4	C19orf52, LDLR, SMARCA4, YIPF2	0.000

SNP, single nucleotide polymorphism; Chr, chromosome; OA, osteoarthritis; PP4, posterior probability for H4 (a joint causal variant) from colocalization analysis.

FA, fatty acid; TotFA, total fatty acid; SFA, saturated fatty acid; MUFA, monounsaturated fatty acid; PUFA, polyunsaturated fatty acid.

Supplementary Table 8. Results of colocalization analysis of pleiotropic loci between fatty acid and OA ($p_{12} = 1 \times 10^{-5}$).

FA	Index SNP	Number of SNPs	PP0	PP1	PP2	PP3	PP4
TotFA	rs10251697	2319	0.001	0.715	2.40E-04	0.202	0.081
	rs10932008	1719	1.55E-07	0.001	1.98E-04	0.999	2.21E-05
	rs1126513	8039	1.41E-11	0.044	3.07E-10	0.956	1.13E-04
	rs113565903	2198	2.78E-04	0.209	5.16E-05	0.038	0.753
	rs11690912	3063	1.83E-16	0.801	4.45E-17	0.195	0.004
	rs12983316	2632	1.83E-56	1.86E-05	9.85E-52	1.000	2.22E-07
	rs13107325	2497	4.52E-12	3.03E-13	0.058	0.003	0.939
	rs1999659	2609	4.80E-05	0.043	0.001	0.953	0.003
	rs2965169	3152	2.70E-119	0.001	3.18E-119	4.93E-04	0.998
	rs429358	3114	2.70E-119	0.001	3.21E-119	0.001	0.998
	rs62033403	2818	0.001	9.84E-05	0.471	0.055	0.473
	rs62101766	3218	2.04E-23	0.925	1.36E-24	0.062	0.013
	rs687339	1511	0.003	0.037	0.032	0.355	0.572
	rs7412	3115	2.70E-119	0.001	3.21E-119	0.001	0.998
	rs7841093	3821	0.002	0.030	0.057	0.908	0.004
	rs79598313	1549	3.41E-05	0.316	6.15E-06	0.056	0.628
	rs9394283	2504	8.09E-09	0.050	1.20E-07	0.735	0.215
	rs9469089	5740	2.82E-10	0.861	4.47E-11	0.137	0.002
SFA	rs10932008	1719	4.41E-05	0.001	0.056	0.941	0.001
	rs113565903	2198	0.001	0.209	9.38E-05	0.038	0.753
	rs115785198	3155	5.55E-83	0.001	6.53E-83	4.95E-04	0.998
	rs11690912	3063	2.70E-15	0.801	6.58E-16	0.195	0.004
	rs13107325	2497	6.65E-13	1.23E-13	0.009	0.001	0.991
	rs2965169	3152	5.55E-83	0.001	6.53E-83	4.94E-04	0.998
	rs429358	3114	5.55E-83	0.001	6.58E-83	0.001	0.998
	rs476330	2612	3.23E-04	0.043	0.007	0.942	0.008
	rs62033403	2818	4.66E-04	1.09E-04	0.261	0.061	0.677
	rs62101766	3218	2.90E-23	0.925	1.94E-24	0.062	0.013
	rs687339	1511	0.007	0.037	0.063	0.356	0.538
	rs7412	3115	5.55E-83	0.001	6.58E-83	0.001	0.998
	rs79598313	1549	1.03E-04	0.316	1.85E-05	0.056	0.627
	rs9394283	2504	8.26E-09	0.050	1.23E-07	0.735	0.216
MUFA	rs1126513	8039	2.22E-12	0.044	4.81E-11	0.956	1.67E-04
	rs11690912	3063	9.16E-10	0.801	2.23E-10	0.195	0.004
	rs1563353	2886	0.003	3.69E-04	0.714	0.086	0.197
	rs193084249	1936	0.014	0.325	0.003	0.076	0.582
	rs34421088	3653	1.35E-04	0.531	1.19E-04	0.467	0.002
	rs41288799	1732	1.94E-149	0.708	7.93E-150	0.289	0.003
	rs4252548	3668	0.009	0.739	0.003	0.216	0.034
	rs429358	3114	2.49E-69	0.001	2.95E-69	0.001	0.998
	rs58132661	3224	2.49E-69	0.001	2.97E-69	0.001	0.998
	rs687339	1511	1.16E-05	0.035	1.11E-04	0.334	0.631
	rs6913037	2470	0.002	0.057	0.018	0.682	0.241
	rs7412	3115	2.49E-69	0.001	2.95E-69	0.001	0.998
	rs7813718	4090	0.105	0.025	0.528	0.126	0.216
	rs7841093	3821	3.19E-05	0.032	0.001	0.960	0.007
	rs9302635	2384	4.25E-04	0.831	7.60E-05	0.149	0.019
	rs9469089	5740	4.45E-11	0.860	7.06E-12	0.136	0.003
	rs10251697	2319	0.002	0.750	4.26E-04	0.212	0.035

PUFA	rs10932008	1718	8.89E-11	0.001	1.14E-07	0.999	0.000
	rs112563428	2390	6.69E-11	0.043	1.18E-09	0.763	0.193
	rs113565903	2198	0.001	0.208	1.74E-04	0.038	0.753
	rs11690912	3063	5.34E-17	0.801	1.30E-17	0.195	0.004
	rs11774915	4202	3.84E-15	0.051	7.15E-14	0.949	2.11E-04
	rs12983316	2632	3.24E-85	1.86E-05	1.74E-80	1.000	2.25E-07
	rs13107325	2497	2.36E-12	1.82E-13	0.030	0.001	0.968
	rs210157	4836	0.011	0.027	0.249	0.642	0.071
	rs28399637	3216	7.20E-309	0.064	8.57E-309	0.075	0.862
	rs3822856	2622	5.42E-07	0.043	1.20E-05	0.951	0.006
	rs429358	3114	7.23E-309	0.064	8.57E-309	0.075	0.862
	rs6060660	1827	7.32E-09	6.61E-07	0.011	0.987	0.003
	rs62033403	2818	4.01E-04	1.09E-04	0.225	0.061	0.714
	rs62101766	3218	1.48E-39	0.925	9.91E-41	0.062	0.013
	rs7248558	2513	3.24E-85	1.86E-05	1.74E-80	1.000	2.25E-07
	rs7412	3115	7.23E-309	0.064	8.57E-309	0.075	0.862
	rs79598313	1549	7.67E-05	0.316	1.38E-05	0.056	0.628
	rs9394283	2504	7.59E-11	0.049	1.13E-09	0.731	0.220
	rs9469089	5740	5.37E-10	0.861	8.52E-11	0.137	0.003

PP0, posterior probability for H0; PP1, posterior probability for H1; PP2, posterior probability for H2; PP3, posterior probability for H3; PP4, posterior probability for H4. OA, osteoarthritis; FA, fatty acid; SNP, single nucleotide polymorphism; TotFA, total fatty acid; SFA, saturated fatty acid; MUFA, monounsaturated fatty acid; PUFA, polyunsaturated fatty acid.

Supplementary Table 9. Robustness of colocalization results ($p_{12} = 5 \times 10^{-6}$).

FA	Index SNP	Number of SNPs	PP0	PP1	PP2	PP3	PP4
TotFA	rs10251697	2319	0.001	0.746	2.51E-04	0.211	0.042
	rs10932008	1719	1.55E-07	0.001	1.98E-04	0.999	1.10E-05
	rs1126513	8039	1.41E-11	0.044	3.07E-10	0.956	5.65E-05
	s11356590[illegible]	2198	4.46E-04	0.335	8.28E-05	0.061	0.604
	rs11690912	3063	1.83E-16	0.803	4.46E-17	0.195	0.002
	rs12983316	2632	1.83E-56	1.86E-05	9.85E-52	1.000	1.11E-07
	rs13107325	2497	8.52E-12	5.70E-13	0.110	0.006	0.885
	rs1999659	2609	4.81E-05	0.043	0.001	0.954	0.001
	rs2965169	3152	5.40E-119	0.003	6.36E-119	9.84E-04	0.996
	rs429358	3114	5.40E-119	0.003	6.40E-119	0.001	0.996
	rs62033403	2818	0.001	1.29E-04	0.617	0.072	0.310
	rs62101766	3218	2.05E-23	0.931	1.37E-24	0.062	0.006
	rs687339	1511	0.005	0.052	0.045	0.497	0.401
	rs7412	3115	5.40E-119	0.003	6.40E-119	0.001	0.996
	rs7841093	3821	0.002	0.030	0.057	0.910	0.002
	rs79598313	1549	4.97E-05	0.460	8.97E-06	0.082	0.458
	rs9394283	2504	9.06E-09	0.056	1.34E-07	0.824	0.120
	rs9469089	5740	2.82E-10	0.862	4.48E-11	0.137	0.001
SFA	rs10932008	1719	4.42E-05	0.001	0.056	0.942	0.001
	s11356590[illegible]	2198	0.001	0.335	1.50E-04	0.061	0.603
	s11578519[illegible]	3155	1.11E-82	0.003	1.30E-82	9.87E-04	0.996
	rs11690912	3063	2.71E-15	0.803	6.59E-16	0.195	0.002
	rs13107325	2497	1.32E-12	2.43E-13	0.017	0.001	0.982
	rs2965169	3152	1.11E-82	0.003	1.30E-82	9.87E-04	0.996
	rs429358	3114	1.11E-82	0.003	1.31E-82	0.001	0.996
	rs476330	2612	3.25E-04	0.043	0.007	0.946	0.004
	rs62033403	2818	7.04E-04	1.65E-04	0.395	0.092	0.512
	rs62101766	3218	2.92E-23	0.931	1.95E-24	0.062	0.006
	rs687339	1511	0.009	0.051	0.086	0.486	0.368
	rs7412	3115	1.11E-82	0.003	1.31E-82	0.001	0.996
	rs79598313	1549	1.50E-04	0.461	2.70E-05	0.082	0.457
	rs9394283	2504	9.26E-09	0.056	1.37E-07	0.824	0.121
MUFA	rs1126513	8039	2.22E-12	0.044	4.81E-11	0.956	8.34E-05
	rs11690912	3063	9.18E-10	0.803	2.23E-10	0.195	0.002
	rs1563353	2886	0.003	4.10E-04	0.791	0.095	0.109
	s19308424[illegible]	1936	0.019	0.459	0.005	0.108	0.410
	rs34421088	3653	1.35E-04	0.532	1.19E-04	0.467	0.001
	rs41288799	1732	1.94E-149	0.709	7.94E-150	0.290	0.002
	rs4252548	3668	0.009	0.751	0.003	0.219	0.017
	rs429358	3114	4.97E-69	0.003	5.90E-69	0.001	0.996
	rs58132661	3224	4.98E-69	0.003	5.93E-69	0.001	0.996
	rs687339	1511	1.70E-05	0.051	1.62E-04	0.488	0.461
	rs6913037	2470	0.002	0.065	0.021	0.776	0.137
	rs7412	3115	4.97E-69	0.003	5.90E-69	0.001	0.996

	rs7813718	4090	0.118	0.028	0.592	0.141	0.121
	rs7841093	3821	3.20E-05	0.032	0.001	0.964	0.004
	rs9302635	2384	4.29E-04	0.840	7.67E-05	0.150	0.010
	rs9469089	5740	4.45E-11	0.862	7.07E-12	0.137	0.002
PUFA	rs10251697	2319	0.002	0.764	4.34E-04	0.216	0.018
	rs10932008	1718	8.89E-11	0.001	1.14E-07	0.999	0.000
	s11256342[illegible]	2390	7.41E-11	0.048	1.30E-09	0.845	0.107
	s11356590[illegible]	2198	0.001	0.334	2.79E-04	0.061	0.604
	rs11690912	3063	5.35E-17	0.803	1.30E-17	0.195	0.002
	rs11774915	4202	3.84E-15	0.051	7.15E-14	0.949	1.05E-04
	rs12983316	2632	3.24E-85	1.86E-05	1.74E-80	1.000	1.12E-07
	rs13107325	2497	4.58E-12	3.54E-13	0.059	0.003	0.938
	rs210157	4836	0.011	0.028	0.258	0.666	0.037
	rs28399637	3216	1.26E-308	0.112	1.51E-308	0.132	0.757
	rs3822856	2622	5.43E-07	0.043	1.20E-05	0.954	0.003
	rs429358	3114	1.27E-308	0.112	1.51E-308	0.131	0.757
	rs6060660	1827	7.33E-09	6.62E-07	0.011	0.988	0.001
	rs62033403	2818	6.23E-04	1.70E-04	0.350	0.094	0.555
	rs62101766	3218	1.49E-39	0.931	9.97E-41	0.062	0.006
	rs7248558	2513	3.24E-85	1.86E-05	1.74E-80	1.000	1.12E-07
	rs7412	3115	1.27E-308	0.112	1.51E-308	0.131	0.757
	rs79598313	1549	1.12E-04	0.460	2.02E-05	0.082	0.458
	rs9394283	2504	8.52E-11	0.055	1.26E-09	0.821	0.124
	rs9469089	5740	5.38E-10	0.862	8.53E-11	0.137	0.001

PP0, posterior probability for H0; PP1, posterior probability for H1; PP2, posterior probability for H2; PP3, posterior probability for H3; PP4, posterior probability for H4. OA, osteoarthritis; FA, fatty acid; SNP, single nucleotide polymorphism; TotFA, total fatty acid; SFA, saturated fatty acid; MUFA, monounsaturated fatty acid; PUFA, polyunsaturated fatty acid.

Supplementary Table 10. Genome-wide gene-based association study was performed in MAGMA based on the GWAS meta-analysis of TotFA and OA.

Gene name	CHR	Start bp	Stop bp	TotFA					OA				
				nSNPs	nParam	N	Z	*P*	nSNPs	nParam	N	Z	*P*
**Known genes in single trait**													
APOE	19	45409039	45412650	13	8	136016	6.11	5.00E-10	15	10	826690	4.48	3.70E-06
DNM2	19	10828729	10944169	496	66	136016	5.95	1.32E-09	639	79	826690	3.17	7.62E-04
FRK	6	116262691	116397360	495	56	136016	6.57	2.47E-11	631	73	826690	3.13	8.76E-04
ILF3	19	10764937	10803095	126	34	136016	4.43	4.66E-06	185	51	826690	6.11	5.00E-10
ZNF76	6	35226691	35263762	165	36	136016	6.84	4.06E-12	228	53	826690	3.61	1.54E-04
DEF6	6	35265595	35289548	103	28	136016	6.89	2.80E-12	132	36	826690	3.41	3.26E-04
WDR12	2	203745323	203776949	118	25	136016	3.17	7.52E-04	151	36	826690	5.61	1.00E-08
DOCK6	19	11309969	11373168	214	41	136016	7.40	6.89E-14	319	55	826690	3.21	6.62E-04
NBEAL1	2	203879597	204091101	544	58	136016	3.44	2.86E-04	709	70	826690	5.37	3.91E-08
ICA1L	2	203637873	203736708	319	29	136016	3.10	9.79E-04	403	41	826690	6.11	5.00E-10
C20orf173	20	34108569	34117481	26	9	136016	3.34	4.22E-04	35	14	826690	6.04	7.73E-10
SCUBE3	6	35181824	35220856	120	33	136016	6.78	6.18E-12	178	57	826690	3.68	1.18E-04
C19orf38	19	10947304	10980466	94	30	136016	6.39	8.17E-11	134	40	826690	3.46	2.71E-04
**Novel shared genes**													
GATA4	8	11534433	11617510	500	77	136016	3.59	1.66E-04	629	81	826690	3.14	8.53E-04
TCP11	6	35085848	35109187	108	19	136016	3.65	1.33E-04	144	30	826690	3.48	2.53E-04
RBM39	20	34291531	34330258	156	17	136016	3.51	2.25E-04	197	25	826690	4.38	5.84E-06
ANKS1A	6	34857038	35085802	908	72	136016	3.61	1.54E-04	1277	93	826690	3.80	7.30E-05
NUDCD3	7	44421965	44530474	359	25	136016	3.80	7.23E-05	439	32	826690	3.23	6.19E-04
VPS4A	16	69345242	69358951	65	20	136016	3.73	9.42E-05	91	31	826690	3.17	7.52E-04
NIP7	16	69373415	69377014	17	8	136016	3.65	1.33E-04	25	13	826690	3.14	8.49E-04
ERGIC3	20	34129778	34145405	49	12	136016	4.17	1.50E-05	51	13	826690	4.78	8.76E-07
CYP2W1	7	1020100	1029276	49	18	136016	4.29	8.83E-06	69	29	826690	3.47	2.61E-04
PINX1	8	10622473	10697394	582	34	136016	3.83	6.42E-05	711	43	826690	3.98	3.43E-05
SLC44A2	19	10713121	10755235	187	34	136016	4.89	5.07E-07	253	35	826690	4.54	2.83E-06
QTRT1	19	10812112	10824043	43	15	136016	5.18	1.10E-07	58	23	826690	4.61	2.00E-06
COG8	16	69362524	69373526	34	12	136016	3.54	2.02E-04	57	24	826690	4.05	2.58E-05
UTP4	16	69166499	69202937	110	41	136016	3.57	1.78E-04	152	59	826690	3.17	7.55E-04
KDF1	1	27276047	27286901	22	9	136016	4.21	1.26E-05	38	16	826690	3.17	7.55E-04
ROMO1	20	34287232	34288902	5	3	136016	3.35	4.07E-04	6	3	826690	4.91	4.46E-07
TMED6	16	69377149	69385712	29	15	136016	4.02	2.86E-05	44	24	826690	3.72	9.77E-05

CHR, chromosome; Start bp, start position; Stop bp, stop position; TotFA, total fatty acid; OA, osteoarthritis.

Supplementary Table 11. Genome-wide gene-based association study was performed in MAGMA based on the GWAS meta-analysis of SFA and OA.

Gene name	CHR	Start bp	Stop bp	SFA					OA				
				nSNPs	nParam	N	Z	*P*	nSNPs	nParam	N	Z	*P*
**Known genes in single trait**													
APOE	19	45409039	45412650	13	8	136016	7.72	6.05E-15	15	10	826690	4.48	3.70E-06
FRK	6	116262691	116397360	496	57	136016	5.77	3.88E-09	631	73	826690	3.13	8.76E-04
ILF3	19	10764937	10803095	126	34	136016	3.91	4.68E-05	185	51	826690	6.11	5.00E-10
ZNF76	6	35226691	35263762	164	35	136016	6.89	2.78E-12	228	53	826690	3.61	1.54E-04
DEF6	6	35265595	35289548	103	28	136016	6.84	4.07E-12	132	36	826690	3.41	3.26E-04
DOCK6	19	11309969	11373168	216	42	136016	6.82	4.49E-12	319	55	826690	3.21	6.62E-04
SCUBE3	6	35181824	35220856	120	33	136016	6.77	6.27E-12	178	57	826690	3.68	1.18E-04
C19orf38	19	10947304	10980466	94	30	136016	6.11	5.00E-10	134	40	826690	3.46	2.71E-04
**Novel shared genes**													
DNM2	19	10828729	10944169	495	66	136016	5.06	2.13E-07	639	79	826690	3.17	7.62E-04
TCP11	6	35085848	35109187	108	19	136016	3.34	4.26E-04	144	30	826690	3.48	2.53E-04
RBM39	20	34291531	34330258	154	16	136016	3.21	6.73E-04	197	25	826690	4.38	5.84E-06
ANKS1A	6	34857038	35085802	907	71	136016	3.23	6.27E-04	1277	93	826690	3.80	7.30E-05
NUDCD3	7	44421965	44530474	358	25	136016	3.71	1.05E-04	439	32	826690	3.23	6.19E-04
VPS4A	16	69345242	69358951	65	20	136016	3.44	2.94E-04	91	31	826690	3.17	7.52E-04
NIP7	16	69373415	69377014	17	8	136016	3.13	8.84E-04	25	13	826690	3.14	8.49E-04
ERGIC3	20	34129778	34145405	49	12	136016	3.60	1.58E-04	51	13	826690	4.78	8.76E-07
CYP2W1	7	1020100	1029276	49	18	136016	3.84	6.05E-05	69	29	826690	3.47	2.61E-04
PINX1	8	10622473	10697394	583	35	136016	3.78	7.79E-05	711	43	826690	3.98	3.43E-05
SLC44A2	19	10713121	10755235	187	34	136016	4.20	1.36E-05	253	35	826690	4.54	2.83E-06
QTRT1	19	10812112	10824043	43	15	136016	4.26	1.02E-05	58	23	826690	4.61	2.00E-06
COG8	16	69362524	69373526	34	12	136016	3.19	7.01E-04	57	24	826690	4.05	2.58E-05
KDF1	1	27276047	27286901	22	9	136016	4.32	7.97E-06	38	16	826690	3.17	7.55E-04
TMED6	16	69377149	69385712	29	15	136016	3.59	1.62E-04	44	24	826690	3.72	9.77E-05

CHR, chromosome; Start bp, start position; Stop bp, stop position; SFA, saturated fatty acid; OA, osteoarthritis.

Supplementary Table 12. Genome-wide gene-based association study was performed in MAGMA based on the GWAS meta-analysis of MUFA and OA.

Gene name	CHR	Start bp	Stop bp	MUFA					OA				
				nSNPs	nParam	N	Z	*P*	nSNPs	nParam	N	Z	*P*
**Known genes in single trait**													
APOE	19	45409039	45412650	13	8	136016	7.20	3.02E-13	15	10	826690	4.48	3.70E-06
**Novel shared genes**													
DNM2	19	10828729	10944169	496	66	136016	4.23	1.16E-05	639	79	826690	3.17	7.62E-04
FRK	6	116262691	116397360	494	55	136016	3.77	8.22E-05	631	73	826690	3.13	8.76E-04
GATA4	8	11534433	11617510	500	77	136016	4.14	1.71E-05	629	81	826690	3.14	8.53E-04
ZNF76	6	35226691	35263762	164	35	136016	4.69	1.35E-06	228	53	826690	3.61	1.54E-04
NUDCD3	7	44421965	44530474	358	25	136016	3.12	9.08E-04	439	32	826690	3.23	6.19E-04
DEF6	6	35265595	35289548	103	28	136016	4.74	1.05E-06	132	36	826690	3.41	3.26E-04
NIP7	16	69373415	69377014	17	8	136016	3.21	6.62E-04	25	13	826690	3.14	8.49E-04
CYP2W1	7	1020100	1029276	49	18	136016	3.65	1.30E-04	69	29	826690	3.47	2.61E-04
PINX1	8	10622473	10697394	582	34	136016	5.14	1.35E-07	711	43	826690	3.98	3.43E-05
SLC44A2	19	10713121	10755235	187	34	136016	3.37	3.80E-04	253	35	826690	4.54	2.83E-06
DOCK6	19	11309969	11373168	215	42	136016	4.97	3.37E-07	319	55	826690	3.21	6.62E-04
QTRT1	19	10812112	10824043	43	15	136016	4.10	2.03E-05	58	23	826690	4.61	2.00E-06
KDF1	1	27276047	27286901	22	9	136016	3.84	6.19E-05	38	16	826690	3.17	7.55E-04
SCUBE3	6	35181824	35220856	121	34	136016	4.67	1.48E-06	178	57	826690	3.68	1.18E-04
C19orf38	19	10947304	10980466	94	30	136016	4.38	5.85E-06	134	40	826690	3.46	2.71E-04
XKR6	8	10753654	11058875	1737	73	136016	4.42	4.91E-06	2320	84	826690	5.19	1.04E-07

CHR, chromosome; Start bp, start position; Stop bp, stop position; MUFA, monounsaturated fatty acid; OA, osteoarthritis.

Supplementary Table 13. Genome-wide gene-based association study was performed in MAGMA based on the GWAS meta-analysis of PUFA and OA.

Gene name	CHR	Start bp	Stop bp	PUFA					OA				
				nSNPs	nParam	N	Z	*P*	nSNPs	nParam	N	Z	*P*
**Known genes in both traits**													
ILF3	19	10764937	10803095	127	34	136016	6.11	5.00E-10	185	51	826690	6.11	5.00E-10
**Known genes in single trait**													
APOE	19	45409039	45412650	13	8	136016	6.11	5.00E-10	15	10	826690	4.48	3.70E-06
DNM2	19	10828729	10944169	496	66	136016	7.39	7.57E-14	639	79	826690	3.17	7.62E-04
FRK	6	116262691	116397360	496	55	136016	6.83	4.27E-12	631	73	826690	3.13	8.76E-04
ZNF76	6	35226691	35263762	165	36	136016	6.11	5.00E-10	228	53	826690	3.61	1.54E-04
CPNE1	20	34213953	34252878	122	16	136016	3.95	3.90E-05	140	19	826690	6.15	3.83E-10
NFS1	20	34256610	34287287	116	24	136016	3.50	2.31E-04	148	36	826690	6.11	5.00E-10
RBM12	20	34236847	34252878	53	11	136016	3.84	6.05E-05	61	14	826690	6.13	4.27E-10
CEP250	20	34042988	34105360	186	25	136016	3.85	5.98E-05	223	35	826690	6.11	5.00E-10
DEF6	6	35265595	35289548	103	28	136016	7.44	4.97E-14	132	36	826690	3.41	3.26E-04
WDR12	2	203745323	203776949	118	25	136016	4.18	1.48E-05	151	36	826690	5.61	1.00E-08
SLC44A2	19	10713121	10755235	187	34	136016	6.15	3.96E-10	253	35	826690	4.54	2.83E-06
DOCK6	19	11309969	11373168	214	41	136016	7.83	2.39E-15	319	55	826690	3.21	6.62E-04
NBEAL1	2	203879597	204091101	544	58	136016	3.68	1.15E-04	709	70	826690	5.37	3.91E-08
CARF	2	203776978	203851060	208	24	136016	3.91	4.55E-05	266	26	826690	5.48	2.16E-08
QTRT1	19	10812112	10824043	43	15	136016	6.08	5.85E-10	58	23	826690	4.61	2.00E-06
ICA1L	2	203637873	203736708	317	29	136016	3.99	3.36E-05	403	41	826690	6.11	5.00E-10
C20orf173	20	34108569	34117481	26	9	136016	3.91	4.63E-05	35	14	826690	6.04	7.73E-10
SCUBE3	6	35181824	35220856	121	34	136016	7.30	1.42E-13	178	57	826690	3.68	1.18E-04
C19orf38	19	10947304	10980466	93	29	136016	7.85	2.16E-15	134	40	826690	3.46	2.71E-04
**Novel shared genes**													
COMP	19	18893583	18902114	24	13	136016	3.97	3.60E-05	33	19	826690	3.16	7.99E-04
GATA4	8	11534433	11617510	499	80	136016	3.19	7.21E-04	629	81	826690	3.14	8.53E-04
TCP11	6	35085848	35109187	108	19	136016	4.39	5.78E-06	144	30	826690	3.48	2.53E-04

VIM	10	17269934	17279592	26	11	136016	3.27	5.30E-04	37	18	826690	3.83	6.36E-05
CDK2AP1	12	123745517	123756863	42	14	136016	3.68	1.15E-04	60	20	826690	3.70	1.08E-04
RBM39	20	34291531	34330258	157	17	136016	4.68	1.40E-06	197	25	826690	4.38	5.84E-06
ANKS1A	6	34857038	35085802	910	73	136016	4.51	3.29E-06	1277	93	826690	3.80	7.30E-05
NUDCD3	7	44421965	44530474	359	25	136016	3.46	2.68E-04	439	32	826690	3.23	6.19E-04
VPS4A	16	69345242	69358951	65	20	136016	3.80	7.35E-05	91	31	826690	3.17	7.52E-04
PHF20	20	34359923	34538292	667	33	136016	3.90	4.87E-05	805	39	826690	5.05	2.24E-07
NIP7	16	69373415	69377014	17	8	136016	3.81	6.97E-05	25	13	826690	3.14	8.49E-04
ERGIC3	20	34129778	34145405	49	12	136016	4.75	1.03E-06	51	13	826690	4.78	8.76E-07
ALKBH5	17	18086867	18113268	121	25	136016	3.68	1.19E-04	147	32	826690	3.78	7.92E-05
CYP2W1	7	1020100	1029276	49	18	136016	4.57	2.40E-06	69	29	826690	3.47	2.61E-04
TTC31	2	74710200	74721691	21	11	136016	3.66	1.24E-04	38	20	826690	3.17	7.62E-04
ILRUN	6	34555065	34664625	459	45	136016	3.16	7.85E-04	632	57	826690	4.19	1.42E-05
COG8	16	69362524	69373526	34	12	136016	3.59	1.63E-04	57	24	826690	4.05	2.58E-05
UTP4	16	69166499	69202937	110	41	136016	3.63	1.42E-04	152	59	826690	3.17	7.55E-04
KDF1	1	27276047	27286901	22	9	136016	3.54	2.04E-04	38	16	826690	3.17	7.55E-04
ROMO1	20	34287232	34288902	5	3	136016	4.66	1.55E-06	6	3	826690	4.91	4.46E-07
CNBD2	20	34556529	34618622	156	28	136016	3.38	3.68E-04	203	39	826690	5.21	9.50E-08
TMED6	16	69377149	69385712	29	15	136016	4.37	6.24E-06	44	24	826690	3.72	9.77E-05
HORMAD2	22	30476453	30573064	285	34	136016	3.59	1.65E-04	344	47	826690	3.78	7.96E-05
FAM171A2	17	42431101	42441235	23	13	136016	3.24	6.02E-04	34	20	826690	3.13	8.88E-04
KMT5A	12	123868704	123893900	86	22	136016	3.93	4.26E-05	114	30	826690	3.77	8.25E-05

CHR, chromosome; Start bp, start position; Stop bp, stop position; PUFA, polyunsaturated fatty acid; OA, osteoarthritis.

Supplementary Table 14. Details of instrument variables for TotFA.

SNP	CHR	Position	TotFA								OA					
			Effect allele	Other allele	Effect allele frequency	Beta	Standard error	*P*-value	R^2	*F*	Effect allele	Other allele	Effect allele frequency	Beta	Standard error	*P*-value
rs1007205	1	62936912	T	C	0.341	-0.086	0.004	5.10E-92	1.75E-03	238.09	T	C	0.344	0.011	0.005	1.96E-02
rs10455872	6	161010118	A	G	0.930	0.073	0.009	2.26E-17	8.64E-04	117.65	A	G	0.926	0.013	0.009	1.38E-01
rs115594766	19	11190110	A	G	0.884	0.105	0.007	1.11E-57	1.13E-03	153.84	A	G	0.887	-0.011	0.007	1.36E-01
rs11591147	1	55505647	T	G	0.029	-0.215	0.015	5.41E-49	5.03E-04	68.49	T	G	0.016	0.023	0.018	2.17E-01
rs116843064	19	8429323	A	G	0.021	-0.104	0.015	1.53E-11	4.77E-04	64.93	A	G	0.022	0.014	0.016	3.91E-01
rs11693150	2	203496575	A	C	0.490	-0.028	0.004	2.52E-12	1.83E-03	250.00	A	C	0.496	-0.005	0.005	2.79E-01
rs11749783	5	74626082	T	C	0.587	-0.045	0.004	1.79E-28	1.83E-03	250.00	T	C	0.623	0.007	0.005	1.15E-01
rs11751024	6	32586236	A	C	0.392	0.036	0.004	7.80E-17	1.71E-03	232.55	A	C	0.396	0.000	0.005	9.84E-01
rs12369145	12	125240154	A	G	0.876	0.039	0.006	1.64E-09	1.15E-03	156.25	A	G	0.906	0.025	0.008	1.69E-03
rs1260326	2	27730940	T	C	0.382	0.095	0.004	3.20E-114	1.75E-03	238.09	T	C	0.388	-0.005	0.005	2.45E-01
rs13240065	7	73015369	A	G	0.126	-0.069	0.006	6.44E-29	1.18E-03	161.29	A	G	0.127	0.003	0.007	6.70E-01
rs139974673	15	44027885	T	C	0.974	-0.145	0.014	1.26E-23	5.10E-04	69.44	T	C	0.973	0.019	0.014	1.81E-01
rs143415093	6	90967337	A	C	0.004	0.320	0.051	2.47E-10	1.45E-04	19.76	A	C	0.003	0.022	0.046	6.42E-01
rs1800588	15	58723675	T	C	0.233	0.131	0.005	5.21E-170	1.56E-03	212.76	T	C	0.214	-0.002	0.005	7.27E-01
rs181948526	4	73768622	T	G	0.016	0.352	0.033	9.33E-27	2.23E-04	30.39	T	G	0.004	-0.285	0.127	2.55E-02
rs1883711	20	39179822	C	G	0.044	0.114	0.012	2.60E-23	6.39E-04	86.96	C	G	0.037	-0.002	0.013	8.56E-01
rs2287997	16	72140553	A	G	0.210	0.031	0.005	4.54E-10	1.47E-03	200.00	A	G	0.188	0.011	0.006	5.70E-02
rs2331415	4	69349677	A	C	0.238	0.043	0.005	4.80E-18	1.47E-03	200.00	A	C	0.244	-0.002	0.005	7.78E-01
rs2575876	9	107665739	A	G	0.242	-0.054	0.005	3.48E-29	1.53E-03	208.33	A	G	0.249	0.009	0.005	7.68E-02
rs2954021	8	126482077	A	G	0.489	0.079	0.004	5.61E-82	1.79E-03	243.90	A	G	0.484	-0.004	0.004	4.22E-01
rs325	8	19819328	T	C	0.901	0.093	0.007	2.44E-41	1.06E-03	144.93	T	C	0.901	-0.003	0.008	7.08E-01
rs34468875	2	21383353	T	C	0.398	0.066	0.004	3.41E-60	1.83E-03	250.00	T	C	0.435	-0.010	0.005	2.78E-02
rs41279633	7	44580876	T	G	0.171	0.033	0.005	6.77E-10	1.36E-03	185.18	T	G	0.163	0.009	0.006	1.40E-01
rs427248	19	49241015	T	C	0.446	0.025	0.004	5.44E-10	1.79E-03	243.90	T	C	0.484	-0.009	0.005	5.05E-02
rs429358	19	45411941	T	C	0.843	-0.131	0.006	1.78E-123	1.33E-03	181.82	T	C	0.845	0.031	0.006	6.08E-07
rs4418728	10	94839724	T	G	0.476	-0.025	0.004	1.01E-09	1.79E-03	243.90	T	G	0.452	0.005	0.005	3.13E-01
rs442177	4	88030261	T	G	0.565	0.027	0.004	8.31E-12	1.83E-03	250.00	T	G	0.587	0.001	0.005	7.75E-01
rs465500	22	21798907	A	G	0.327	-0.029	0.004	2.98E-11	1.71E-03	232.55	A	G	0.307	0.004	0.005	3.87E-01
rs4876611	8	116671848	A	G	0.287	-0.030	0.004	3.02E-11	1.67E-03	227.27	A	G	0.282	-0.006	0.005	2.62E-01
rs58542926	19	19379549	T	C	0.071	-0.137	0.008	4.96E-67	9.30E-04	126.58	T	C	0.076	-0.003	0.009	7.01E-01
rs59950280	4	3452345	A	G	0.316	0.032	0.004	2.32E-13	1.67E-03	227.27	A	G	0.331	-0.008	0.005	1.05E-01
rs646776	1	109818530	T	C	0.777	0.065	0.005	7.54E-43	1.53E-03	208.33	T	C	0.779	-0.014	0.005	1.07E-02
rs6699113	1	25777743	T	C	0.531	0.026	0.004	2.84E-10	1.79E-03	243.90	T	C	0.546	-0.003	0.004	5.18E-01
rs684818	1	234854779	T	C	0.547	0.027	0.004	1.07E-11	1.83E-03	250.00	T	C	0.528	0.008	0.005	6.93E-02
rs6882345	5	156397673	A	G	0.645	0.043	0.004	6.34E-26	1.79E-03	243.90	A	G	0.636	-0.005	0.005	3.41E-01
rs7140110	13	114544024	T	C	0.703	-0.031	0.005	6.18E-12	1.63E-03	222.22	T	C	0.703	0.003	0.005	5.95E-01
rs72875462	2	44079310	A	C	0.070	-0.073	0.008	5.82E-19	8.96E-04	121.95	A	C	0.061	0.016	0.009	1.00E-01
rs73066485	7	21611970	T	G	0.791	-0.036	0.005	3.59E-13	1.50E-03	204.08	T	G	0.783	0.010	0.005	8.23E-02
rs7310615	12	111865049	C	G	0.452	-0.027	0.004	1.81E-10	1.75E-03	238.09	C	G	0.471	-0.004	0.005	3.82E-01
rs7609911	3	57713360	A	C	0.557	-0.025	0.004	3.84E-10	1.83E-03	250.00	A	C	0.572	0.004	0.005	3.87E-01
rs77960347	18	47109955	A	G	0.988	-0.225	0.020	7.07E-30	3.71E-04	50.50	A	G	0.987	0.016	0.020	4.15E-01
rs79202680	17	4692640	T	G	0.032	-0.146	0.019	8.99E-15	3.91E-04	53.19	T	G	0.005	-0.064	0.034	6.08E-02
rs79598313	1	27284913	T	C	0.030	0.083	0.013	1.20E-10	5.70E-04	77.52	T	C	0.025	-0.051	0.015	7.65E-04
rs8205	6	35263677	A	T	0.813	-0.038	0.005	1.00E-12	1.39E-03	188.68	A	T	0.818	-0.023	0.006	1.52E-04
rs964184	11	116648917	C	G	0.850	-0.182	0.006	8.58E-222	1.29E-03	175.44	C	G	0.865	0.014	0.007	3.70E-02

CHR, chromosome; SNP, single nucleotide polymorphism; TotFA, total fatty acid; OA, osteoarthritis.

Supplementary Table 15. Details of instrument variables for SFA.

SNP	CHR	Position	SFA								OA					
			Effect allele	Other allele	Effect allele frequency	Beta	Standard error	*P*-value	R^2	*F*	Effect allele	Other allele	Effect allele frequency	Beta	Standard error	*P*-value
rs1007205	1	62936912	T	C	0.340	-0.077	0.004	5.97E-74	1.75E-03	238.09	T	C	0.344	0.011	0.005	1.96E-02
rs10455872	6	161010118	A	G	0.930	0.076	0.009	5.70E-19	8.54E-04	116.28	A	G	0.926	0.013	0.009	1.38E-01
rs115594766	19	11190110	A	G	0.884	0.090	0.007	3.47E-43	1.13E-03	153.84	A	G	0.887	-0.011	0.007	1.36E-01
rs11591147	1	55505647	T	G	0.029	-0.191	0.015	4.09E-39	5.03E-04	68.49	T	G	0.016	0.023	0.018	2.17E-01
rs11749783	5	74626082	T	C	0.586	-0.042	0.004	3.53E-25	1.79E-03	243.90	T	C	0.623	0.007	0.005	1.15E-01
rs1260326	2	27730940	T	C	0.382	0.086	0.004	3.57E-94	1.75E-03	238.09	T	C	0.388	-0.005	0.005	2.45E-01
rs12997242	2	21381177	A	G	0.397	0.057	0.004	1.92E-45	1.79E-03	243.90	A	G	0.436	-0.010	0.005	2.94E-02
rs139974673	15	44027885	T	C	0.974	-0.145	0.014	8.84E-24	5.10E-04	69.44	T	C	0.973	0.019	0.014	1.81E-01
rs143415093	6	90967337	A	C	0.004	0.314	0.051	5.03E-10	1.46E-04	19.80	A	C	0.003	0.022	0.046	6.42E-01
rs17231506	16	56994528	T	C	0.315	0.027	0.004	2.58E-10	1.71E-03	232.55	T	C	0.323	0.000	0.005	9.57E-01
rs174574	11	61600342	A	C	0.358	-0.039	0.004	3.97E-21	1.75E-03	238.09	A	C	0.357	-0.006	0.005	1.85E-01
rs1800588	15	58723675	T	C	0.233	0.130	0.005	3.89E-167	1.56E-03	212.76	T	C	0.214	-0.002	0.005	7.27E-01
rs181948526	4	73768622	T	G	0.016	0.284	0.033	6.34E-18	2.23E-04	30.39	T	G	0.004	-0.285	0.127	2.55E-02
rs1883711	20	39179822	C	G	0.044	0.101	0.012	1.07E-18	6.39E-04	86.96	C	G	0.037	-0.002	0.013	8.56E-01
rs2331415	4	69349677	A	C	0.238	0.041	0.005	4.58E-16	1.47E-03	200.00	A	C	0.244	-0.002	0.005	7.78E-01
rs2524137	6	31264582	T	C	0.710	0.035	0.005	1.29E-14	1.63E-03	222.22	T	C	0.694	-0.003	0.006	5.56E-01
rs2575876	9	107665739	A	G	0.242	-0.048	0.005	7.35E-24	1.53E-03	208.33	A	G	0.249	0.009	0.005	7.68E-02
rs2954021	8	126482077	A	G	0.489	0.076	0.004	2.02E-75	1.79E-03	243.90	A	G	0.484	-0.004	0.004	4.22E-01
rs325	8	19819328	T	C	0.901	0.083	0.007	4.73E-33	1.06E-03	144.93	T	C	0.901	-0.003	0.008	7.08E-01
rs427248	19	49241015	T	C	0.446	0.028	0.004	1.56E-11	1.79E-03	243.90	T	C	0.484	-0.009	0.005	5.05E-02
rs429358	19	45411941	T	C	0.843	-0.110	0.006	4.82E-87	1.33E-03	181.82	T	C	0.845	0.031	0.006	6.08E-07
rs4418728	10	94839724	T	G	0.475	-0.025	0.004	8.00E-10	1.83E-03	250.00	T	G	0.452	0.005	0.005	3.13E-01
rs442177	4	88030261	T	G	0.565	0.025	0.004	3.02E-10	1.83E-03	250.00	T	G	0.587	0.001	0.005	7.75E-01
rs465500	22	21798907	A	G	0.327	-0.027	0.004	3.13E-10	1.71E-03	232.55	A	G	0.307	0.004	0.005	3.87E-01
rs4738684	8	59393273	A	G	0.349	0.027	0.004	3.18E-10	1.71E-03	232.55	A	G	0.337	-0.001	0.005	8.52E-01
rs58542926	19	19379549	T	C	0.071	-0.116	0.008	1.57E-48	9.30E-04	126.58	T	C	0.076	-0.003	0.009	7.01E-01
rs59950280	4	3452345	A	G	0.316	0.027	0.004	8.29E-10	1.67E-03	227.27	A	G	0.331	-0.008	0.005	1.05E-01
rs646776	1	109818530	T	C	0.777	0.054	0.005	1.31E-29	1.53E-03	208.33	T	C	0.779	-0.014	0.005	1.07E-02
rs6874202	5	156391628	T	C	0.355	-0.038	0.004	1.10E-19	1.79E-03	243.90	T	C	0.363	0.005	0.005	3.27E-01
rs72875462	2	44079310	A	C	0.070	-0.067	0.008	3.87E-16	8.85E-04	120.48	A	C	0.061	0.016	0.009	1.00E-01
rs73066485	7	21611970	T	G	0.791	-0.035	0.005	7.90E-13	1.50E-03	204.08	T	G	0.783	0.010	0.005	8.23E-02
rs77960347	18	47109955	A	G	0.988	-0.225	0.020	1.02E-29	3.71E-04	50.50	A	G	0.987	0.016	0.020	4.15E-01
rs79202680	17	4692640	T	G	0.032	-0.143	0.019	2.61E-14	3.91E-04	53.19	T	G	0.005	-0.064	0.034	6.08E-02
rs79598313	1	27284913	T	C	0.030	0.080	0.013	4.39E-10	5.70E-04	77.52	T	C	0.025	-0.051	0.015	7.65E-04
rs80189144	7	72939939	T	C	0.878	0.056	0.006	5.05E-19	1.18E-03	161.29	T	C	0.879	-0.007	0.007	3.20E-01
rs9380497	6	35161629	A	G	0.189	0.037	0.005	7.28E-13	1.44E-03	196.08	A	G	0.182	0.022	0.006	2.15E-04
rs9604489	13	114542957	C	G	0.743	-0.038	0.006	7.63E-10	1.20E-03	163.93	C	G	0.739	0.004	0.006	5.41E-01
rs964184	11	116648917	C	G	0.850	-0.158	0.006	2.22E-167	1.29E-03	175.44	C	G	0.865	0.014	0.007	3.70E-02

CHR, chromosome; SNP, single nucleotide polymorphism; SFA, saturated fatty acid; OA, osteoarthritis.

Sanderson-Windmeijer conditional F-statistic = 163.35

Supplementary Table 16. Details of instrument variables for MUFA.

SNP	CHR	Position	MUFA								OA					
			Effect allele	Other allele	Effect allele frequency	Beta	Standard error	*P*-value	R^2	*F*	Effect allele	Other allele	Effect allele frequency	Beta	Standard error	*P*-value
rs1007205	1	62936912	T	C	0.340	-0.072	0.004	7.53E-65	1.75E-03	238.09	T	C	0.344	0.011	0.005	1.96E-02
rs10455872	6	161010118	A	G	0.930	0.102	0.009	1.13E-32	8.54E-04	116.28	A	G	0.926	0.013	0.009	1.38E-01
rs115594766	19	11190110	A	G	0.884	0.077	0.007	1.23E-31	1.13E-03	153.84	A	G	0.887	-0.011	0.007	1.36E-01
rs11591147	1	55505647	T	G	0.029	-0.135	0.015	3.26E-20	5.03E-04	68.49	T	G	0.016	0.023	0.018	2.17E-01
rs116843064	19	8429323	A	G	0.021	-0.163	0.016	3.96E-26	4.74E-04	64.52	A	G	0.022	0.014	0.016	3.91E-01
rs11693150	2	203496575	A	C	0.490	-0.025	0.004	7.31E-10	1.83E-03	250.00	A	C	0.496	-0.005	0.005	2.79E-01
rs11749783	5	74626082	T	C	0.586	-0.032	0.004	1.72E-15	1.79E-03	243.90	T	C	0.623	0.007	0.005	1.15E-01
rs1260326	2	27730940	T	C	0.382	0.112	0.004	1.69E-156	1.75E-03	238.09	T	C	0.388	-0.005	0.005	2.45E-01
rs13225450	7	72976123	T	C	0.122	-0.091	0.006	2.98E-48	1.18E-03	161.29	T	C	0.122	0.004	0.007	5.36E-01
rs139315015	8	19893297	A	G	0.895	0.126	0.007	5.15E-77	1.08E-03	147.06	A	G	0.897	0.000	0.008	9.89E-01
rs139974673	15	44027885	T	C	0.974	-0.165	0.014	2.50E-30	5.10E-04	69.44	T	C	0.973	0.019	0.014	1.81E-01
rs174553	11	61575158	A	G	0.647	-0.046	0.004	3.58E-28	1.75E-03	238.09	A	G	0.650	0.006	0.005	2.15E-01
rs1800588	15	58723675	T	C	0.233	0.118	0.005	2.12E-138	1.56E-03	212.76	T	C	0.214	-0.002	0.005	7.27E-01
rs181948526	4	73768622	T	G	0.016	0.308	0.033	7.60E-21	2.23E-04	30.39	T	G	0.004	-0.285	0.127	2.55E-02
rs1883711	20	39179822	C	G	0.044	0.096	0.012	6.85E-17	6.39E-04	86.96	C	G	0.037	-0.002	0.013	8.56E-01
rs2074493	6	31239776	A	C	0.716	-0.040	0.005	1.15E-18	1.63E-03	222.22	A	C	0.756	-0.013	0.006	1.91E-02
rs2287997	16	72140553	A	G	0.211	0.031	0.005	6.55E-10	1.47E-03	200.00	A	G	0.188	0.011	0.006	5.70E-02
rs2331415	4	69349677	A	C	0.238	0.040	0.005	1.03E-15	1.47E-03	200.00	A	C	0.244	-0.002	0.005	7.78E-01
rs2645429	8	11660051	A	G	0.248	0.030	0.005	2.22E-10	1.53E-03	208.33	A	G	0.246	-0.001	0.005	9.13E-01
rs2740488	9	107661742	A	C	0.748	0.047	0.005	5.89E-23	1.56E-03	212.76	A	C	0.738	-0.008	0.005	1.13E-01
rs2954021	8	126482077	A	G	0.489	0.085	0.004	1.41E-94	1.79E-03	243.90	A	G	0.484	-0.004	0.004	4.22E-01
rs427248	19	49241015	T	C	0.447	0.028	0.004	9.94E-12	1.79E-03	243.90	T	C	0.484	-0.009	0.005	5.05E-02
rs429358	19	45411941	T	C	0.843	-0.100	0.006	2.45E-73	1.33E-03	181.82	T	C	0.845	0.031	0.006	6.08E-07
rs4296389	2	21142994	T	C	0.312	-0.064	0.005	3.38E-44	1.60E-03	217.39	T	C	0.258	-0.002	0.005	7.03E-01
rs442177	4	88030261	T	G	0.565	0.032	0.004	2.48E-15	1.83E-03	250.00	T	G	0.587	0.001	0.005	7.75E-01
rs458361	22	21798351	C	G	0.674	0.027	0.004	4.93E-10	1.71E-03	232.55	C	G	0.693	-0.004	0.005	4.57E-01
rs4715317	6	52629010	T	G	0.354	0.027	0.004	2.17E-10	1.71E-03	232.55	T	G	0.350	-0.003	0.005	5.19E-01
rs4876611	8	116671848	A	G	0.287	-0.030	0.005	4.90E-11	1.63E-03	222.22	A	G	0.282	-0.006	0.005	2.62E-01
rs56130071	7	21598753	C	G	0.208	0.030	0.005	7.27E-10	1.50E-03	204.08	C	G	0.217	-0.010	0.005	7.38E-02
rs58542926	19	19379549	T	C	0.071	-0.133	0.008	2.31E-63	9.30E-04	126.58	T	C	0.076	-0.003	0.009	7.01E-01
rs59950280	4	3452345	A	G	0.316	0.035	0.004	3.57E-15	1.67E-03	227.27	A	G	0.331	-0.008	0.005	1.05E-01
rs660240	1	109817838	T	C	0.218	-0.046	0.005	6.61E-22	1.53E-03	208.33	T	C	0.214	0.013	0.006	1.65E-02
rs687339	3	135932359	T	C	0.792	0.030	0.005	9.12E-10	1.50E-03	204.08	T	C	0.777	0.024	0.005	1.32E-05
rs6874202	5	156391628	T	C	0.355	-0.041	0.004	5.81E-22	1.75E-03	238.09	T	C	0.363	0.005	0.005	3.27E-01
rs7140110	13	114544024	T	C	0.703	-0.032	0.005	2.74E-12	1.63E-03	222.22	T	C	0.703	0.003	0.005	5.95E-01
rs73079476	12	21343833	A	C	0.833	-0.034	0.006	1.24E-09	1.33E-03	181.82	A	C	0.838	-0.003	0.006	5.87E-01
rs7609911	3	57713360	A	C	0.557	-0.025	0.004	6.31E-10	1.83E-03	250.00	A	C	0.572	0.004	0.005	3.87E-01
rs76866386	2	44075483	T	C	0.928	0.050	0.008	7.70E-10	9.07E-04	123.45	T	C	0.936	-0.014	0.009	1.32E-01
rs77697917	17	41840849	T	C	0.030	0.085	0.013	1.48E-11	5.88E-04	80.00	T	C	0.029	-0.006	0.014	6.46E-01
rs78058190	2	219699999	A	G	0.068	0.062	0.010	3.54E-10	7.42E-04	101.01	A	G	0.065	-0.010	0.011	3.62E-01
rs79202680	17	4692640	T	G	0.032	-0.164	0.019	3.00E-18	3.91E-04	53.19	T	G	0.005	-0.064	0.034	6.08E-02
rs964184	11	116648917	C	G	0.850	-0.197	0.006	4.46E-260	1.29E-03	175.44	C	G	0.865	0.014	0.007	3.70E-02
rs9687846	5	55861894	A	G	0.181	0.034	0.005	5.34E-11	1.41E-03	192.30	A	G	0.190	-0.001	0.006	8.37E-01
rs9958734	18	47118398	T	C	0.886	-0.051	0.008	1.09E-10	9.30E-04	126.58	T	C	0.929	0.009	0.010	3.86E-01
rs998584	6	43757896	A	C	0.492	0.025	0.004	1.39E-09	1.79E-03	243.90	A	C	0.480	-0.008	0.005	9.55E-02

CHR, chromosome; SNP, single nucleotide polymorphism; MUFA, monounsaturated fatty acid; OA, osteoarthritis.

Sanderson-Windmeijer conditional F-statistic = 171.88

Supplementary Table 17. Details of instrument variables for PUFA.

SNP	CHR	Position	PUFA								OA					
			Effect allele	Other allele	Effect allele frequency	Beta	Standard error	*P*-value	R^2	*F*	Effect allele	Other allele	Effect allele frequency	Beta	Standard error	*P*-value
rs10020631	4	69353863	A	G	0.241	0.041	0.005	2.95E-16	1.44E-03	196.08	A	G	0.250	-0.004	0.005	4.75E-01
rs1007205	1	62936912	T	C	0.341	-0.090	0.004	1.48E-102	1.75E-03	238.09	T	C	0.344	0.011	0.005	1.96E-02
rs10184673	2	169827796	A	G	0.568	-0.026	0.004	3.79E-10	1.79E-03	243.90	A	G	0.599	-0.007	0.005	1.41E-01
rs10265736	7	1172465	T	C	0.171	0.036	0.005	3.99E-11	1.36E-03	185.18	T	C	0.169	-0.007	0.006	2.68E-01
rs10283206	8	55410392	T	C	0.215	0.031	0.005	3.17E-10	1.50E-03	204.08	T	C	0.225	0.002	0.005	6.83E-01
rs10793620	10	46087283	A	G	0.687	-0.026	0.004	9.99E-10	1.71E-03	232.55	A	G	0.691	-0.001	0.005	8.07E-01
rs10832963	11	18664241	T	G	0.312	-0.027	0.004	6.06E-10	1.71E-03	232.55	T	G	0.270	0.005	0.005	2.91E-01
rs11170420	12	53504257	T	C	0.126	-0.038	0.006	1.48E-09	1.18E-03	161.29	T	C	0.118	-0.003	0.007	6.57E-01
rs11513245	12	9296354	A	C	0.686	-0.027	0.004	2.83E-10	1.71E-03	232.55	A	C	0.684	-0.006	0.005	2.07E-01
rs115594766	19	11190110	A	G	0.884	0.128	0.007	1.46E-86	1.13E-03	153.84	A	G	0.887	-0.011	0.007	1.36E-01
rs11571725	13	32938881	A	G	0.772	0.029	0.005	1.25E-09	1.53E-03	208.33	A	G	0.779	0.004	0.005	4.78E-01
rs11591147	1	55505647	T	G	0.029	-0.282	0.015	2.41E-83	5.03E-04	68.49	T	G	0.016	0.023	0.018	2.17E-01
rs11693150	2	203496575	A	C	0.490	-0.030	0.004	2.53E-14	1.88E-03	256.41	A	C	0.496	-0.005	0.005	2.79E-01
rs11751024	6	32586236	A	C	0.391	0.035	0.004	2.42E-16	1.71E-03	232.55	A	C	0.396	0.000	0.005	9.84E-01
rs12446515	16	56987015	T	C	0.315	0.052	0.004	5.31E-33	1.71E-03	232.55	T	C	0.323	0.000	0.006	9.73E-01
rs1260326	2	27730940	T	C	0.382	0.068	0.004	9.53E-60	1.75E-03	238.09	T	C	0.388	-0.005	0.005	2.45E-01
rs12916	5	74656539	T	C	0.570	-0.053	0.004	7.51E-41	1.83E-03	250.00	T	C	0.598	0.006	0.005	2.31E-01
rs13240065	7	73015369	A	G	0.126	-0.046	0.006	7.10E-14	1.20E-03	163.93	A	G	0.127	0.003	0.007	6.70E-01
rs142385484	19	50016759	T	C	0.152	-0.037	0.006	1.86E-10	1.27E-03	172.41	T	C	0.156	0.008	0.006	1.88E-01
rs1461729	8	9187242	A	G	0.122	-0.058	0.006	7.11E-20	1.17E-03	158.73	A	G	0.101	0.003	0.008	7.00E-01
rs174574	11	61600342	A	C	0.358	-0.068	0.004	3.22E-61	1.79E-03	243.90	A	C	0.357	-0.006	0.005	1.85E-01
rs1800588	15	58723675	T	C	0.233	0.118	0.005	2.41E-141	1.56E-03	212.76	T	C	0.214	-0.002	0.005	7.27E-01
rs1800961	20	43042364	T	C	0.034	-0.075	0.011	3.72E-11	6.45E-04	87.72	T	C	0.036	-0.003	0.013	7.92E-01
rs181948526	4	73768622	T	G	0.016	0.407	0.033	4.99E-35	2.23E-04	30.39	T	G	0.004	-0.285	0.127	2.55E-02
rs1883711	20	39179822	C	G	0.044	0.122	0.012	2.00E-26	6.39E-04	86.96	C	G	0.037	-0.002	0.013	8.56E-01
rs2244608	12	121416988	A	G	0.648	-0.029	0.004	3.42E-12	1.75E-03	238.09	A	G	0.675	-0.011	0.005	2.33E-02
rs2287997	16	72140553	A	G	0.210	0.032	0.005	5.40E-11	1.50E-03	204.08	A	G	0.188	0.011	0.006	5.70E-02
rs2618566	20	17844684	T	G	0.652	-0.027	0.004	4.95E-10	1.71E-03	232.55	T	G	0.665	-0.003	0.005	5.25E-01
rs2740488	9	107661742	A	C	0.748	0.055	0.005	4.76E-32	1.56E-03	212.76	A	C	0.738	-0.008	0.005	1.13E-01
rs291	8	19815852	T	C	0.768	0.039	0.005	1.52E-15	1.53E-03	208.33	T	C	0.762	0.005	0.005	3.53E-01
rs2954021	8	126482077	A	G	0.489	0.058	0.004	2.43E-46	1.79E-03	243.90	A	G	0.484	-0.004	0.004	4.22E-01
rs41279633	7	44580876	T	G	0.171	0.033	0.005	9.08E-10	1.36E-03	185.18	T	G	0.163	0.009	0.006	1.40E-01
rs465500	22	21798907	A	G	0.327	-0.026	0.004	8.18E-10	1.71E-03	232.55	A	G	0.307	0.004	0.005	3.87E-01
rs4713845	6	35228387	C	G	0.186	0.042	0.005	5.38E-15	1.39E-03	188.68	C	G	0.180	0.023	0.006	1.15E-04
rs484084	1	234857676	T	C	0.453	-0.037	0.004	5.31E-20	1.83E-03	250.00	T	C	0.471	-0.008	0.005	8.24E-02
rs4876611	8	116671848	A	G	0.288	-0.032	0.004	6.82E-13	1.67E-03	227.27	A	G	0.282	-0.006	0.005	2.62E-01
rs548145	2	21291312	T	C	0.181	-0.096	0.005	3.61E-78	1.44E-03	196.08	T	C	0.172	0.006	0.006	3.60E-01
rs56132765	2	44078853	A	G	0.070	-0.089	0.008	1.72E-27	8.96E-04	121.95	A	G	0.061	0.017	0.010	7.78E-02
rs56268840	3	12271381	T	C	0.261	-0.034	0.005	8.79E-13	1.56E-03	212.76	T	C	0.270	0.004	0.005	4.24E-01
rs58542926	19	19379549	T	C	0.072	-0.130	0.008	1.09E-61	9.42E-04	128.20	T	C	0.076	-0.003	0.009	7.01E-01
rs59950280	4	3452345	A	G	0.316	0.029	0.004	3.41E-11	1.67E-03	227.27	A	G	0.331	-0.008	0.005	1.05E-01
rs635634	9	136155000	T	C	0.187	0.046	0.005	1.18E-19	1.47E-03	200.00	T	C	0.190	0.003	0.006	6.68E-01
rs646776	1	109818530	T	C	0.777	0.085	0.005	1.18E-71	1.56E-03	212.76	T	C	0.779	-0.014	0.005	1.07E-02
rs6699113	1	25777743	T	C	0.531	0.030	0.004	1.13E-13	1.79E-03	243.90	T	C	0.546	-0.003	0.004	5.18E-01
rs6882345	5	156397673	A	G	0.645	0.046	0.004	1.43E-29	1.79E-03	243.90	A	G	0.636	-0.005	0.005	3.41E-01
rs7140110	13	114544024	T	C	0.703	-0.028	0.005	2.57E-10	1.63E-03	222.22	T	C	0.703	0.003	0.005	5.95E-01
rs73066485	7	21611970	T	G	0.791	-0.034	0.005	2.45E-12	1.53E-03	208.33	T	G	0.783	0.010	0.005	8.23E-02
rs7310615	12	111865049	C	G	0.452	-0.038	0.004	1.43E-19	1.75E-03	238.09	C	G	0.471	-0.004	0.005	3.82E-01
rs7412	19	45412079	T	C	0.075	-0.302	0.008	0.00E+00	9.30E-04	126.58	T	C	0.080	0.034	0.008	5.76E-05
rs7776156	6	16113321	A	G	0.472	-0.025	0.004	1.76E-09	1.79E-03	243.90	A	G	0.465	-0.004	0.005	4.22E-01
rs77960347	18	47109955	A	G	0.988	-0.283	0.020	2.26E-46	3.71E-04	50.50	A	G	0.987	0.016	0.020	4.15E-01
rs79598313	1	27284913	T	C	0.030	0.081	0.013	2.80E-10	5.74E-04	78.12	T	C	0.025	-0.051	0.015	7.65E-04
rs80254170	6	160514283	A	G	0.926	-0.052	0.008	2.10E-11	9.42E-04	128.20	A	G	0.924	0.007	0.009	4.28E-01
rs9488822	6	116312893	A	T	0.662	0.028	0.004	4.39E-11	1.71E-03	232.55	A	T	0.655	0.010	0.005	3.68E-02
rs964184	11	116648917	C	G	0.850	-0.150	0.006	2.05E-152	1.29E-03	175.44	C	G	0.865	0.014	0.007	3.70E-02

CHR, chromosome; SNP, single nucleotide polymorphism; PUFA, polyunsaturated fatty acid; OA, osteoarthritis.

Sanderson-Windmeijer conditional F-statistic = 165.34

Supplementary Table 18. False discovery rate (FDR)-adjusted *P*-values across MR analyses.

exposure	One-sample_Model1_FDR	One-sample_Model2_FDR	Two-sample_IVW_FDR	Non-linear_Model1_FDR	Non-linear_Model2_FDR
TotFA	0.24	0.18	9.66E-04	0.64	0.80
SFA	0.10	0.08	1.64E-04	0.64	0.80
MUFA	0.10	0.06	2.02E-03	0.64	0.80
PUFA	0.27	0.11	1.71E-03	0.64	0.80

Supplementary Table 19. Two-sample MR sensitivity analyses of fatty acids on OA.

Exposure	Cochran's Q	Q_P	MREgger intercept (95%CI)	Egger_P	MRPRESSO_Global_RSSobs	MRPRESSO_Global_P
TotFA	106.84	3.82E-07	0.003 (-0.002, 0.008)	0.24	111.21	<0.05
SFA	78.85	7.41E-05	-0.001 (-0.006, 0.005)	0.74	83.32	<0.05
MUFA	96.39	8.75E-06	0.000 (-0.004, 0.005)	0.92	100.02	<0.05
PUFA	102.86	6.94E-05	0.006 (0.002, 0.009)	<0.05	107.32	<0.05

Supplementary Table 20. Steiger directionality test results for two-sample MR analyses.

exposure	outcome	R^2.exposure	R^2.outcome	correct_causal_direction	steiger_pval
TotFA	OA	0.05	3.08E-04	TRUE	<0.001
SFA	OA	0.04	2.72E-04	TRUE	<0.001
MUFA	OA	0.05	2.76E-04	TRUE	<0.001
PUFA	OA	0.06	2.83E-04	TRUE	<0.001

Supplementary Table 21. Summary of two-sample MR and sensitivity analyses.

Exposure	nSNPs	Mean F-statistic	R^2	IVW *OR* (95% *CI*)	Weighted median	MR-Egger	MR-Egger intercept	MRPRESSO_Global_*P*	Cochran's Q	lowest leave-one-out *P*	excluding lipid metabolism regions
TotFA	45	178.75	0.06	0.93 (0.89-0.97)	0.93 (0.89-0.97)	0.90 (0.84-0.96)	0.003	<0.05	106.84	4.29E-03	0.94 (0.91-0.98)
SFA	38	173.98	0.05	0.91 (0.88-0.95)	0.92 (0.88-0.97)	0.93 (0.85-1.00)	-0.001	<0.05	78.85	3.46E-04	0.93 (0.89-0.97)
MUFA	45	181.00	0.06	0.94 (0.90-0.98)	0.95 (0.91-0.99)	0.94 (0.87-1.00)	0.000	<0.05	96.39	0.01	0.95 (0.92-0.99)
PUFA	55	193.41	0.08	0.95 (0.92-0.98)	0.92 (0.88-0.95)	0.88 (0.84-0.93)	0.006	<0.05	102.86	0.03	0.96 (0.93-1.00)

Supplementary Table 22. Instrument strength, statistical power and heterogeneity in nonlinear MR analyses.

Exposure	Stratum	Sample size	F-statistic	Causal effect we can detect at 80% power	Heterogeneity_Model1_*P*	Heterogeneity_Model2_*P*
TotFA	Lowest stratum	44606	63.17	< 0.88 or > 1.14	0.69	0.55
	Stratum 2	44605	63.17	< 0.88 or > 1.14		
	Stratum 3	44605	63.17	< 0.88 or > 1.14		
	Highest stratum	44605	63.17	< 0.88 or > 1.14		
SFA	Lowest stratum	43541	60.25	< 0.87 or > 1.15	0.53	0.78
	Stratum 2	43541	60.25	< 0.87 or > 1.15		
	Stratum 3	43540	60.23	< 0.87 or > 1.15		
	Highest stratum	43541	60.25	< 0.87 or > 1.15		
MUFA	Lowest stratum	43449	61.56	< 0.88 or > 1.14	0.10	0.33
	Stratum 2	43448	61.55	< 0.88 or > 1.14		
	Stratum 3	43448	61.55	< 0.88 or > 1.14		
	Highest stratum	43448	61.55	< 0.88 or > 1.14		
PUFA	Lowest stratum	44003	69.48	< 0.90 or > 1.12	0.03	0.93
	Stratum 2	44002	69.46	< 0.90 or > 1.12		
	Stratum 3	44002	69.46	< 0.90 or > 1.12		
	Highest stratum	44002	69.46	< 0.90 or > 1.12		

Supplementary Figure

**Figure**	**Title**
S1	Regional association plots of the APOE locus (tagged by rs429358 and rs7412).
S2	MR estimates excluding the APOE region and pleiotropic outliers.
S3	Leave-one-out sensitivity analysis.
S4	Single-SNP sensitivity analysis.
S5	Multivariable MR analysis between fatty acid and OA.
S6	Reverse MR analysis with OA as the exposure.
S7	Non-linear MR estimates across four population strata in the basic model.
S8	Non-linear MR estimates across four population strata in the fully adjusted model.
S9	Two-sample MR analyses of n-3 and n-6 PUFA in relation to OA.
S10	Two-sample MR analyses in non-overlapping samples.

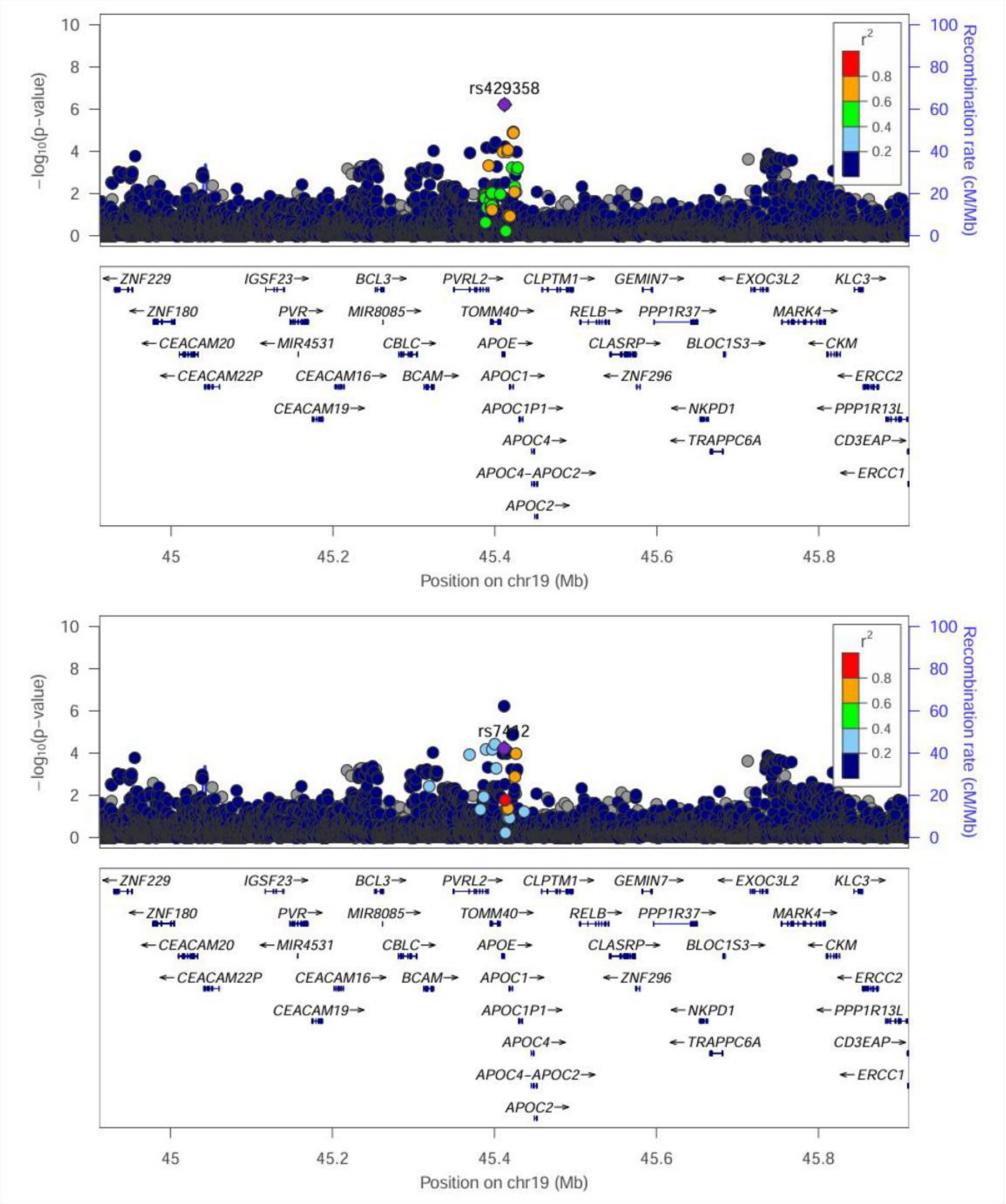

Supplementary Figure 1. Regional association plots of the APOE locus (tagged by rs429358 and rs7412).

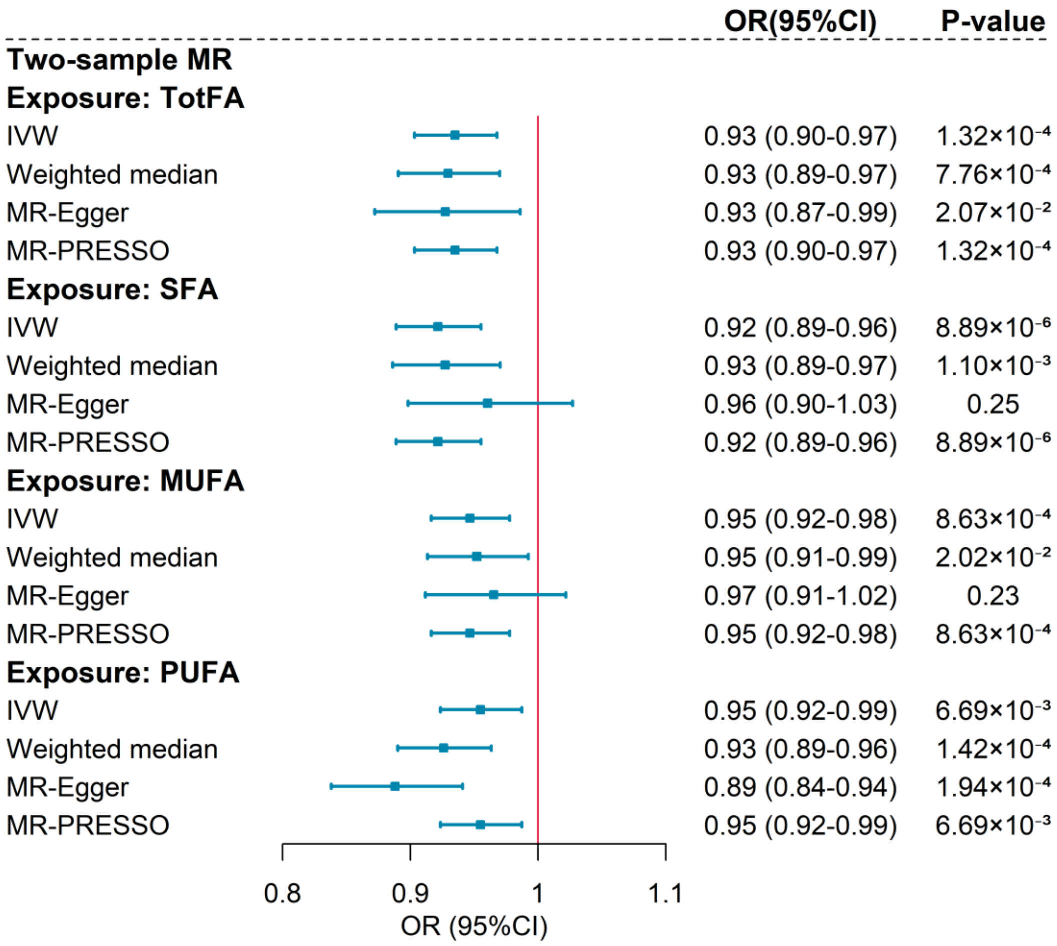

Supplementary Figure 2. MR estimates excluding the APOE region and pleiotropic outliers.

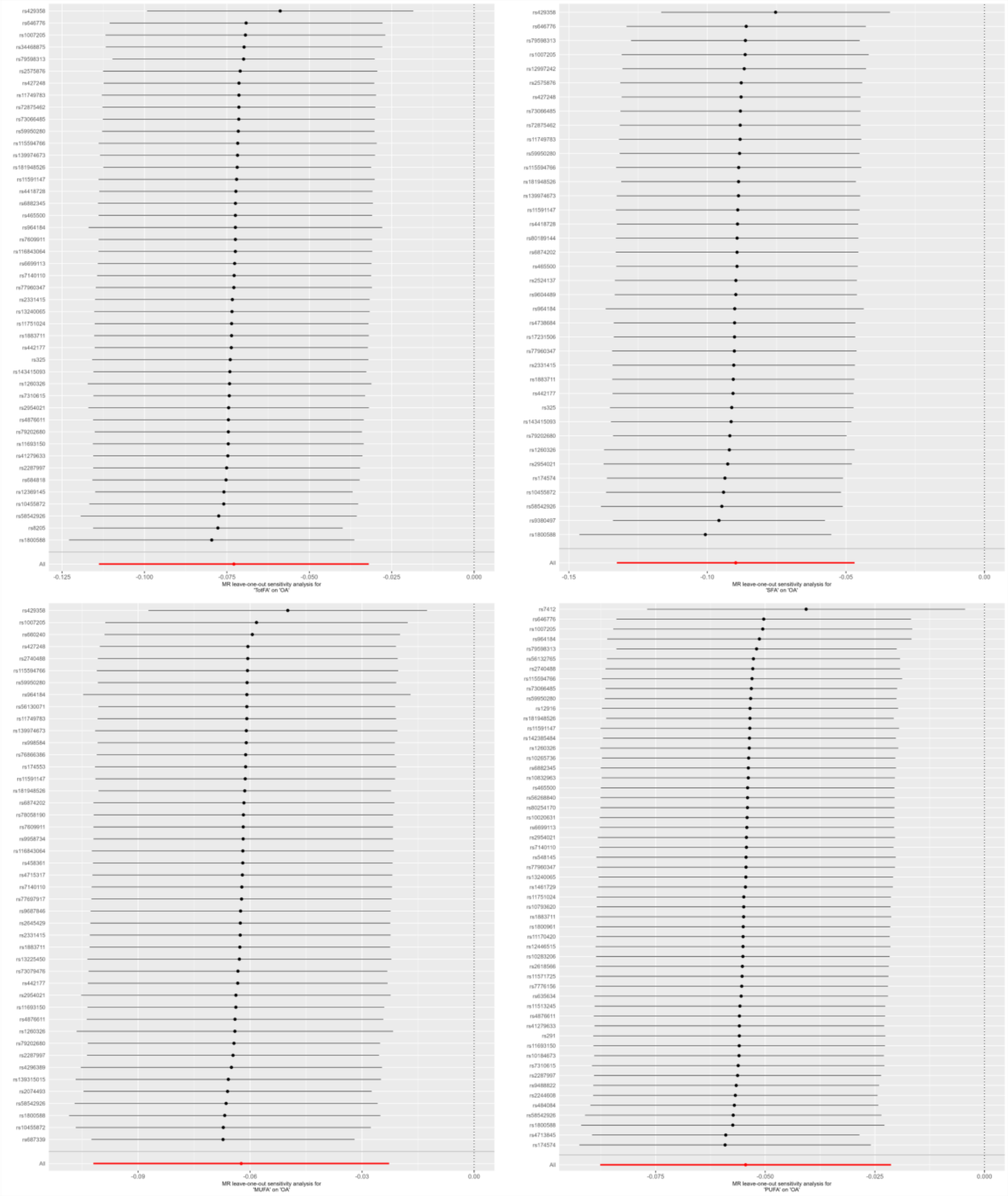

Supplementary Figure 3. Leave-one-out sensitivity analysis.

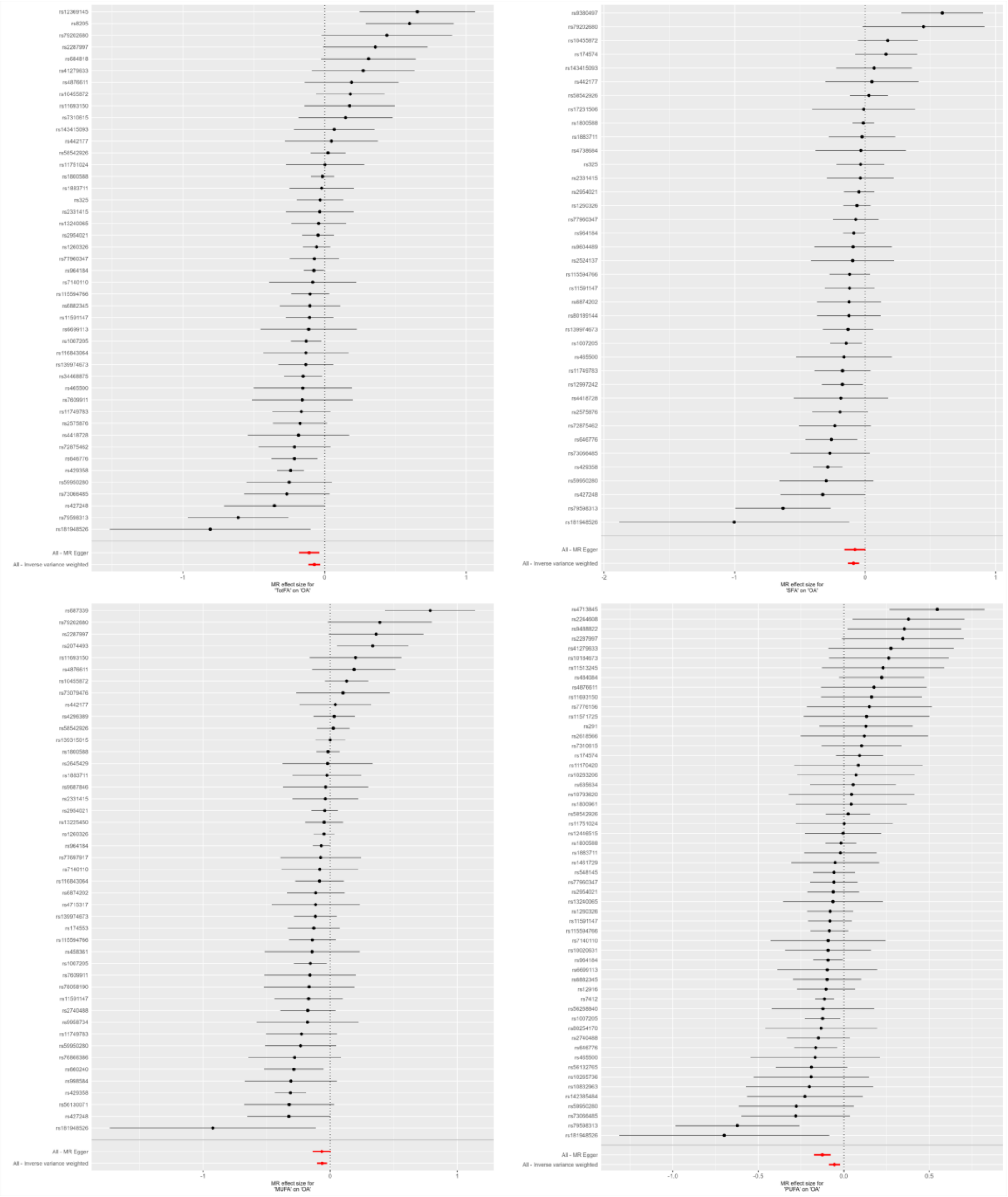

Supplementary Figure 4. Single-SNP sensitivity analysis.

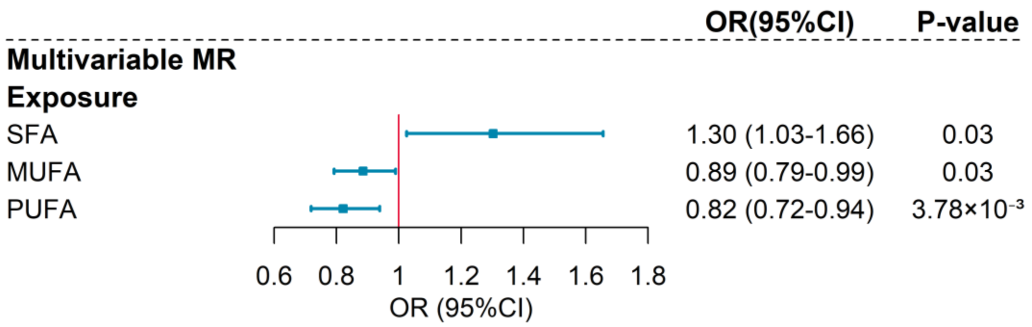

Supplementary Figure 5. Multivariable MR analysis between fatty acid and OA.

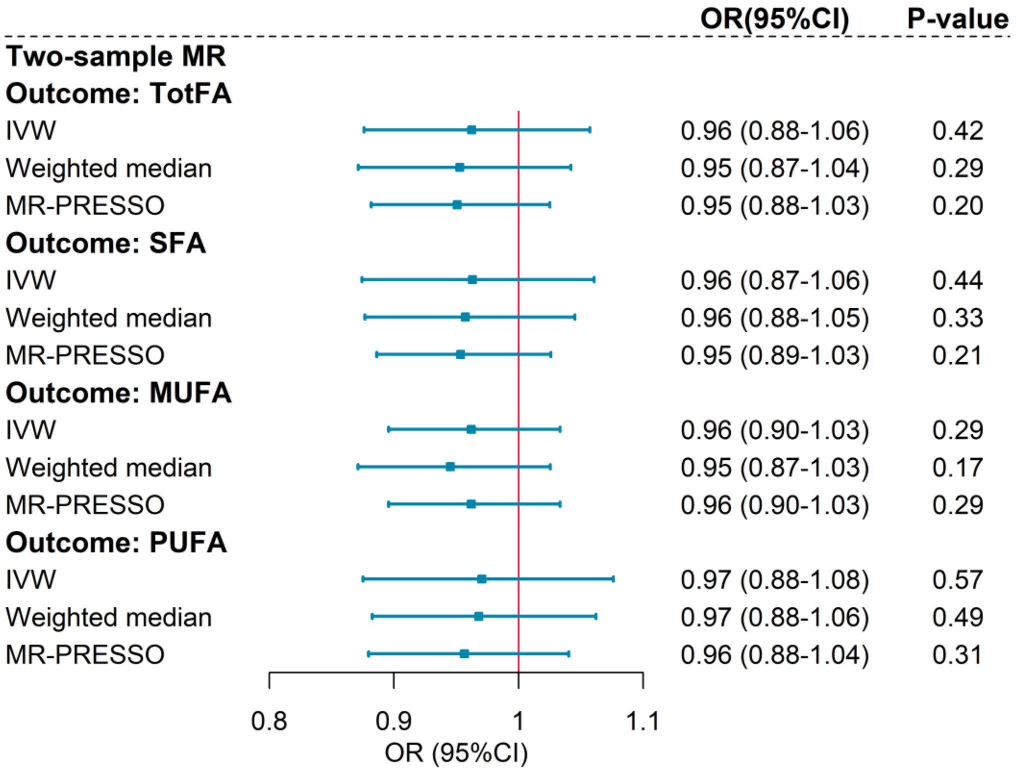

Supplementary Figure 6. Reverse MR analysis with OA as the exposure.

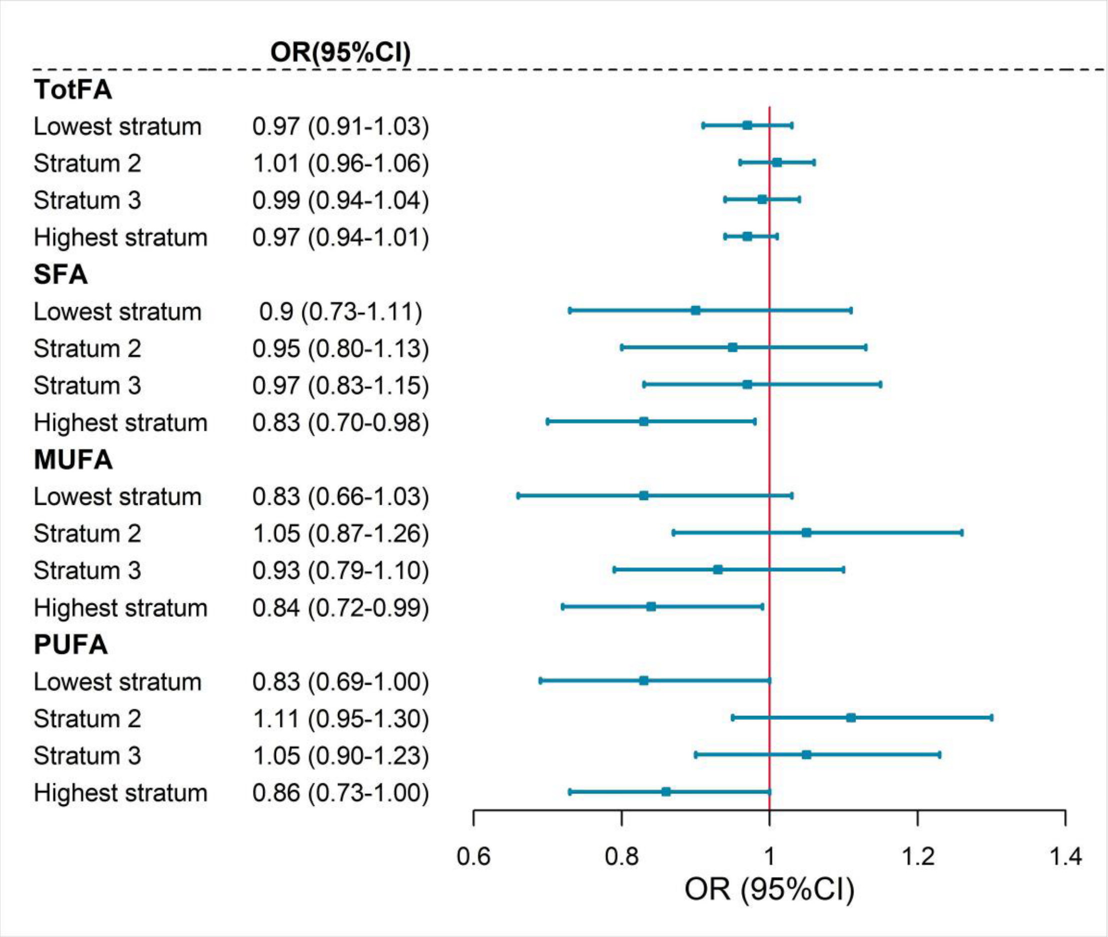

Supplementary Figure 7. Non-linear MR estimates across four population strata in the basic model.

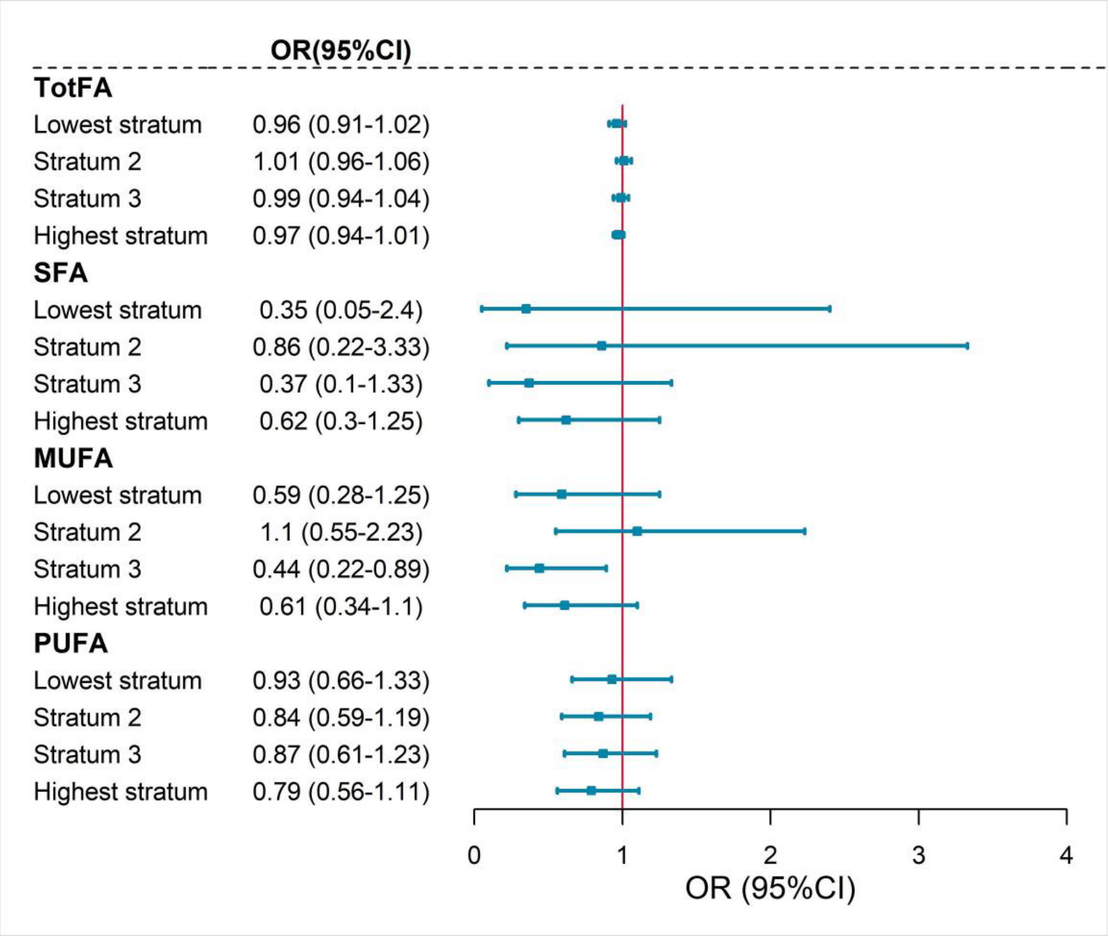

Supplementary Figure 8. Non-linear MR estimates across four population strata in the fully adjusted model.

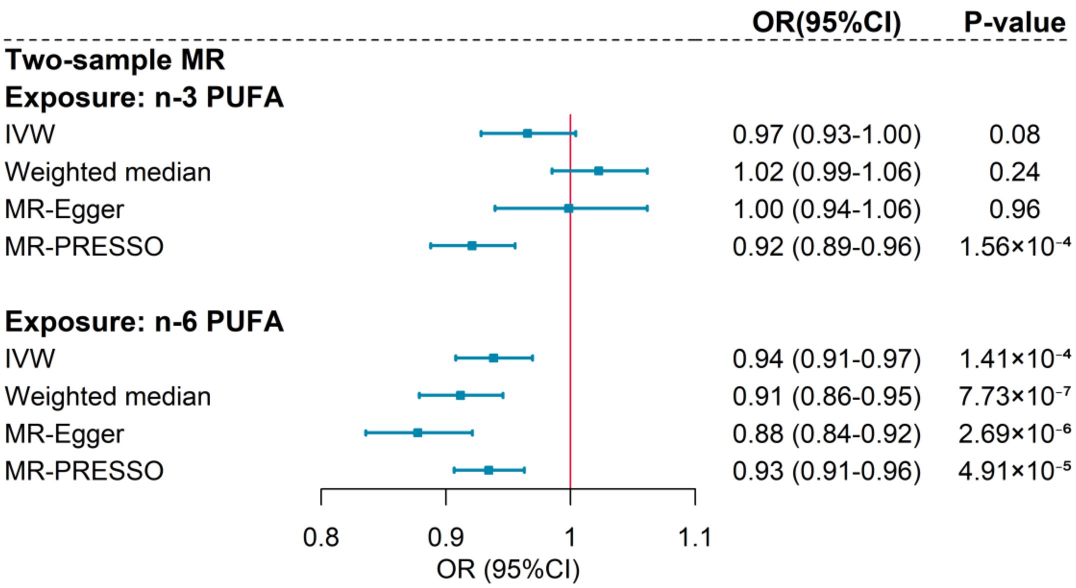

Supplementary Figure 9. Two-sample MR analyses of n-3 and n-6 PUFA in relation to OA.

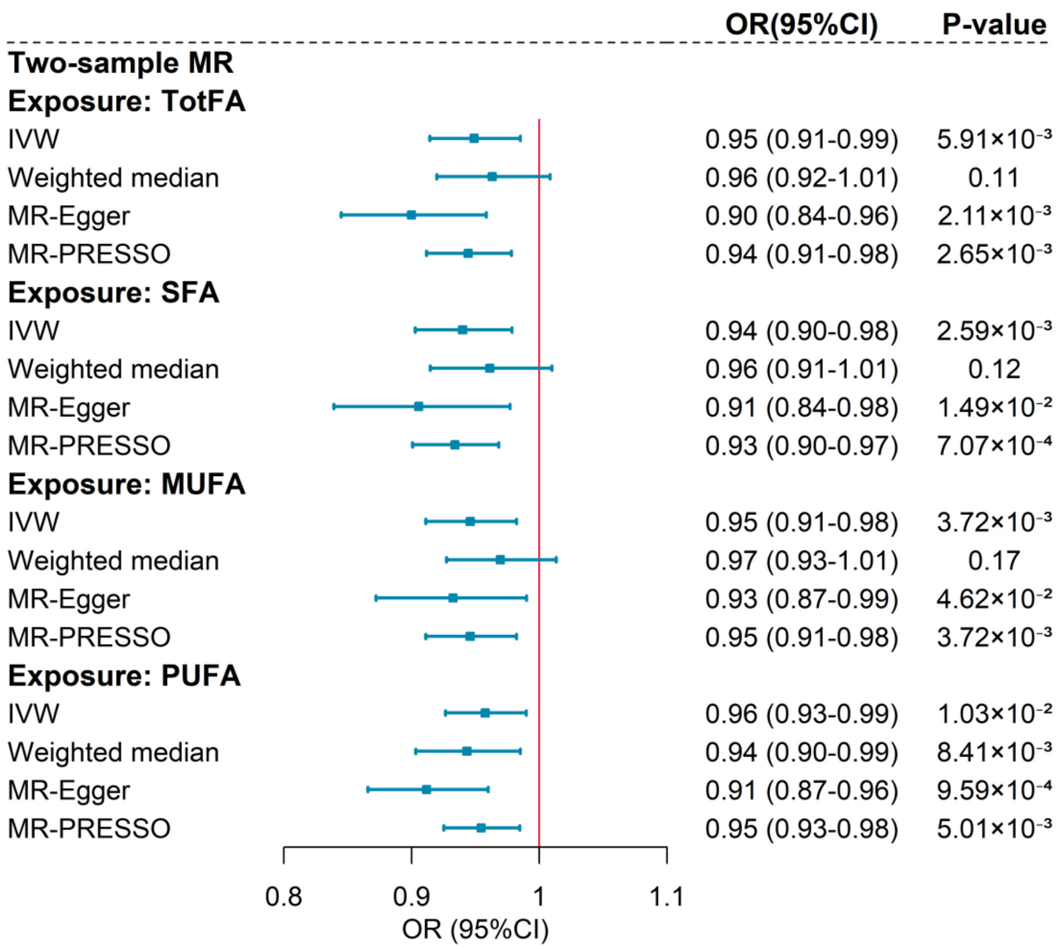

Supplementary Figure 10. Two-sample MR analyses in non-overlapping samples.
